# VAPB confers selective neuroprotection by driving autophagic degradation of pathogenic aggregates in ALS

**DOI:** 10.1186/s40478-026-02298-8

**Published:** 2026-05-29

**Authors:** Priyanka Tripathi, Haihong Guo, Alfred Yamoah, Ramkumar Mathur, Panagiotis Doukas, Alice Dreser, Christopher Marvin Jesse, Eleonora Aronica, Andreas Hermann, Harry Steinbusch, Gary Anthony Brook, Joachim Weis, Anand Goswami

**Affiliations:** 1 Institute of Neuropathology, RWTH Aachen University Hospital, Pauwelsstr. 30, 52074 Aachen, Germany; 2 Department of Geriatrics, School of Medicine and Health Sciences, University of North Dakota, Grand Forks, ND 58202 USA; 3 Amsterdam UMC, Department of (Neuro) Pathology, Amsterdam Neuroscience, University of Amsterdam, Meibergdreef 9, 1105 AZ Amsterdam, The Netherlands; 4 Department of Psychiatry and Neuropsychology, School for Mental Health and Neuroscience, Maastricht University, Maastricht, The Netherlands; 5 Department of Neurosurgery, Inselspital, Bern University Hospital, University of Bern, Bern, Switzerland; 6 Translational Neurodegeneration Section “Albrecht Kossel”, Department of Neurology and Center for Transdisciplinary Neurosciences Rostock (CTNR), University Medical Center Rostock, 18147 Rostock, Germany; 7 German Centre for Neurodegenerative Diseases (DZNE) Rostock/Greifswald, 18147 Rostock, Germany; 8 EURON - European Graduate School of Neuroscience, Maastricht University, Maastricht, The Netherlands; 9 Present Address: Department of Neurology, Center for Motor Neuron Biology and Disease, Columbia University, New York, NY USA; 10 Present Address: Department of Neurology, Eleanor and Lou Gehrig ALS Center, Columbia University, New York, NY USA

**Keywords:** ALS8, VAPB, Autophagy, RBPs, Selective MN vulnerability

## Abstract

During the progression of amyotrophic lateral sclerosis (ALS), only specific motor neurons (MNs) preferentially deteriorate, while others are spared until the disease reaches its end stage. Resilient MNs possess several protective factors, yet the precise molecular mechanism(s) underlying selective neuronal vulnerability remains poorly understood. Vesicle-associated membrane protein (VAMP)-binding protein B (VAPB) is an endoplasmic reticulum (ER) protein involved in protein quality control (PQC) mechanisms, including unfolded protein response (UPR) as well as autophagy. A dominantly inherited P56S mutation in the VAPB gene has been linked to ALS8, atypical ALS, and late-onset spinal muscular atrophy (SMA). The P56S VAPB mutation causes ER-associated inclusions, disorganization, and ER stress, contributing to MN degeneration through toxic gain and loss of function. Over-expression of VAPB protein confers neuroprotection in a mouse model of ALS, and increased levels of neuronal VAPB inversely correlate with the absence of pathological aggregates. We hypothesize that VAPB is crucial for motor neuron survival by promoting autophagic degradation of ALS-associated aggregates, while lack of VAPB confers neuronal vulnerability. We analyzed the brain and spinal cord from sporadic (s) and familial (f) ALS patients, comparing patterns of VAPB immunoreactivity using immunohistochemistry, complemented by Western and dot blot analysis. Pathophysiological insights from these studies were further explored using cell culture models, including MNs derived from induced pluripotent stem cells (iPSCs). Consistent with our hypothesis we observed that MNs/neurons resistant to ALS exhibited elevated levels of VAPB and were devoid of pathogenic aggregates. Similarly, ALS-resistant oculomotor neurons showed increased VAPB immunoreactivity compared to normal controls. VAPB was often found to be sequestered within toxic aggregates alongside autophagy-related proteins in the lumbar spinal cord MNs. Notably, a compensatory increase in VAPB immunoreactivity was observed at the C-bouton synapse, suggesting a potential alternative mechanism of neuroprotection. Supporting these findings, in vitro experiments indicated that VAPB overexpression promoted autophagy and assisted in clearing ALS-associated RNA-binding protein aggregates. In summary, VAPB promotes selective neuronal survival by facilitating the autophagic clearance of toxic aggregates. Abnormal VAPB accumulations likely disrupt these neuroprotective processes.

**Supplementary Information:**

The online version contains supplementary material available at 10.1186/s40478-026-02298-8.

## Introduction

Recent genetic studies have identified mutations in numerous crucial genes that regulate protein quality control (PQC) mechanisms, particularly autophagy and RNA-binding protein (RBP) homeostasis, which are associated with amyotrophic lateral sclerosis (ALS) [42, 55]. Interestingly, many of these genes have been found to promote neuroprotection and enhance the efficient degradation of toxic misfolded proteins and aggregates as part of the proteostasis network [2, 24, 55, 65, 99]. However, despite this, aggregated or misfolded proteins reaching critical levels have been shown to sequester proteins involved in proteostasis networking. This leads to impairment of degradation pathways, resulting in the generation of more toxic aggregates and further exacerbating age-related dysfunction and neurodegeneration [42, 55]. Moreover, disease-specific toxic misfolded proteins trigger defects in numerous RNA/DNA-dependent pathways, encompassing transcriptional abnormalities, nucleocytoplasmic shuttling, stress granule (SGs) dynamics, and DNA damage and repair (DDR) signaling [42, 55, 74]. These insights shed light on the intricate interplay between protein misfolding, RNA/DNA dysregulation, and neurodegeneration in ALS pathology.

It is intriguing that despite the ubiquitous expression of ALS-associated PQC proteins, only a specific subset of motor neurons (MNs) is selectively vulnerable, while others remain protected until the end stage of the disease [81, 82, 98]. Furthermore, these vulnerable neurons exhibit a higher propensity to accumulate disease-associated misfolded proteins, likely due to the absence of neuroprotective factors and other biochemical features [21, 98]. These findings underscore the complexity of cell type-specific pathogenic mechanisms associated with ALS. Understanding the molecular distinctions between vulnerable and resilient MNs could provide valuable insights into ALS and aid in developing effective therapies [8].

Among the PQC genes that have been associated with ALS, a dominantly inherited mutation (P56S) in autophagy-associated vesicle-associated membrane protein-associated protein B (VAPB) has been linked to typical ALS (ALS8), atypical ALS and late-onset spinal muscular atrophy (SMA) [24, 77, 78]. P56S VAPB protein forms endoplasmic reticulum (ER)-associated inclusions and induces toxicity by inducing ER stress and ER disorganization [12, 83, 104]. Recent studies using various cell cultures as well as knockout and knock-in mouse models suggest both toxic gain and loss of VAPB function in MN degeneration [44, 51, 83, 104]. Although over-expression of VAPB was shown to slow motor impairment and neuromuscular denervation in a mouse model of ALS, the mechanism underlying disease progression facilitated by mutant VAPB, and the neuroprotection exerted by Wt-VAPB remains uncertain.

VAPB is an ER membrane-anchored protein and is associated with ER-Golgi intermediate vesicles. It is widely expressed and particularly abundant in the central nervous system [43, 53, 102]. The VAPB protein contains an N-terminal major sperm protein (MSP) domain, housing a putative α-helical coiled-coil and a single transmembrane domain. The MSP domain of VAPB is functionally crucial due to its ability to bind to various proteins containing FFAT motifs (two phenylalanines in an acidic region), enabling the tethering of ER to organelles. These specific interactions orchestrate multiple roles, including the maintenance of ER structure and functions [4, 87], modulation of responses to ER stress [25, 45, 104], facilitating retrograde transport of proteins [102], and lipid transfer to the Golgi apparatus [84]. Additionally, several recent reports have suggested the involvement of ER-VAPB tethering in regulating ER autophagy (ER-phagy) [79], autophagosome biogenesis [114] and the regulation of autophagy in general [27]. Consistent with this, our recent research has demonstrated that VAPB plays a role in ER-orchestrated protein homeostasis and regulation of the fusion of autophagosomes to lysosomes [43, 108], thereby providing a vital foundation for neuronal survival and maintaining neuronal PQC. Neuronal PQC relies on multiple strategies, such as molecular chaperones, autophagy, the ubiquitin–proteasome system, endoplasmic reticulum-associated degradation (ERAD), and the formation of stress granules (SGs) [9, 46] to maintain proteostasis [1, 34, 101, 106]. Since the decline in PQC leads to the aggregation of specific proteins in neurodegenerative diseases, including ALS, it is conceivable that restoring proteostasis by enhancing various PQC mechanisms could prevent, slow down, or even eliminate toxic protein inclusions [1, 22, 34, 37, 106].

Building upon previous concepts and recognizing VAPB’s active involvement in both PQC and autophagy mechanisms, our study aims to elucidate how VAPB contributes to selective neuronal resilience, whether VAPB-mediated autophagy can aid in the removal of toxic aggregates, and how VAPB is embedded in the broader concept of ALS pathology. Given VAPB’s pivotal role in maintaining PQC [45, 63] and considering the dysregulation of proteostasis in ALS [101, 106]**,** we hypothesize that VAPB supports selective neuronal survival by enhancing autophagic clearance of toxic aggregates, and that abnormal VAPB accumulation disrupts these protective mechanisms, contributing to neuronal vulnerability in ALS.

Consistent with our hypothesis, we observed a distinct pattern of VAPB immunoreactivity in cortical neurons and disease-resistant spinal MNs in ALS. This supports both the concept of selective neuronal resistance as well as the failure of PQC in vulnerable neurons. ALS-resistant oculomotor neurons also showed increased levels of VAPB staining, confirming this observation. Furthermore, elevated VAPB immunoreactivity was associated with enlarged C-terminal synapses, suggesting a compensatory role of VAPB in MN protection. Finally, our cell culture experiments further supported our findings, showing that VAPB overexpression promotes the clearance of pathogenic aggregates through autophagy activation. In summary, VAPB enhances neuronal resistance by facilitating the autophagic clearance of toxic aggregates. However, the sequestration of VAPB within aggregates suggests its potential failure to maintain proteostasis/PQC in vulnerable neurons.

## Materials and methods

### Reagents and antibodies

Fluorescent nucleic acid stain Hoechst 33,258 was purchased from Molecular Probes. Thapsigargin, MG132, Rapamycin, Bafilomycin A, protease inhibitor cocktail was purchased from Sigma Aldrich. All primary and secondary antibodies and their dilutions used in this study are listed in Supplementary Table S2. Many of these commercial, previously used antibodies have been validated by us for their consistency both in immunofluorescence (IF) and immunohistochemistry (IHC) and Western blot analysis (WB) in our studies (see references (1–5) in Supplementary Table S1). Rabbit polyclonal VAPB antibody was custom-made and validated for its consistency, both in IF, IHC and WB in previous studies [29, 43, 72, 86].

### Human *post-mortem* tissue

Frozen post-mortem tissue either from frontal cortex (control; n = 3, C9orf72; n = 5) or lumbar spinal cord tissue (control; n = 5, sALS; n = 9, C9orf72; n = 8) (also described for Filter trap assay- FTA, synaptic preparation and Western blot analysis) as well as formalin-fixed paraffin-embedded brain (motor cortex, frontal cortex, midbrain and hippocampus) and lumbar spinal cord sections were obtained from the at Amsterdam UMC, University of Amsterdam (n = 7 sALS patients, n = 5 C9orf72-fALS patients, n = 4 FUS-fALS (R521C) patients, and n = 4 age-matched controls). We examined following areas of brain (motor cortex, frontal cortex, midbrain and hippocampus) and spinal cord (Lumbar spinal cord) because these were are major areas affected in ALS-FTD. Post-mortem tissue was obtained 6–30 h after death (Supplementary Table S1). The number of sections per case used in individual experiments is mentioned in the figure legends. Both the IHC protocols for human and mice samples were performed using similar protocols and were standardized based upon fixation methods and has been extensively reported in previous publications (See Tables). All ALS patients met the El Escorial criteria [58], as independently verified by two neuropathologists. The control group consisted of adults without any history of neurological disease, confirmed by their last clinical evaluation. Demographic details of all ALS and controls patients are summarized in Supplementary Table S1.

### Mouse Tissue

ALS mice expressing high copy numbers of human mutant G93A-SOD1 [31] were used in this study. Lumbar spinal cord tissue from the disease-affected 12-week-old male mice and their corresponding control littermates were used for all experiments (n = 3 for each genotype for WB analysis and n = 3 for each genotype for IHC). FFPE sections and Frozen tissue from Lumbar spinal cord of SOD1 (12-week-old male mice and their corresponding control littermates) were generously provided by Dr. Sonja Johann from the Department of Neuroanatomy (RWTH Aachen) through our established collaboration. The procedures were approved by the Review Board for the Care of Animal Subjects of the district government (North-Rhine Westphalia, Germany), RWTH Aachen University Hospital Institutional Animal Care and Use Committee and performed according to international guidelines on the use of laboratory mice (reference number 84–02.04. 2013. A087; Germany).

### Immunohistochemistry

*Diaminobenzidine (DAB)* 3–4 µm paraffin sections were placed on poly-L-lysine coated slides and allowed to dry in an oven (37 °C) overnight and then processed for immunohistochemistry or other routine staining (H&E, Nissl) are described in detail elsewhere[43]. Sections were deparaffinized in xylene for 20 min, then rehydrated in 100%, 95%, and 70% ethanol for 5 min each. Endogenous peroxidase activity was quenched with 0.3% H_2_O_2_ in methanol for 20 min. Antigen retrieval was performed by heating sections in citrate buffer (pH 6, DAKO) for 20 min in a pressure cooker. After washing in PBS, sections were incubated with the primary antibody (Supplementary Table S2) for 1 h at room temperature or overnight at 4 °C. Following another PBS wash, sections were incubated with a polymeric HRP-linker secondary antibody (IL Immunologic, Duiven, The Netherlands) for 30 min at room temperature. The sections were then stained with DAB reagent (DCS Innovative Diagnostic System DAB kit) for 3–4 min till the brown color appears. For FUS and TDP- 43 antibodies, we standardized the incubation time for 2 min. The reaction was stopped by immersing the sections in distilled water and counterstained with 6% hematoxylin for 3 min. All procedures were conducted at room temperature. Standard histological and histochemical stains, including H & E, were used as described previously [18].

*IF- Staining* Single and double immunofluorescence staining was performed as described elsewhere [17, 43]. In brief, deparaffinized tissue sections were heated in citrate buffer (pH 6, Dako) for 20 min in a pressure cooker for antigen retrieval. Sections were then blocked with ready-to-use 10% normal goat serum (Life Technologies, MD, USA) for 1 h at room temperature to avoid non-specific binding. They were then incubated with the primary antibody at 4 °C overnight. After a 10-min wash in TBS-T, the sections were incubated with an Alexa-conjugated secondary antibody (1:500 in TBS-T) at room temperature for 2 h. Sections were rewashed in TBS-T (2 × 10 min) and stained for 10 min with 0.1% Sudan Black in 80% ethanol to suppress endogenous lipofuscin autofluorescence. Finally, the sections were washed for 5 min in TBS-T and mounted with Vectashield mounting medium (Vector Laboratories) containing DAPI.

**Fig. 1:**
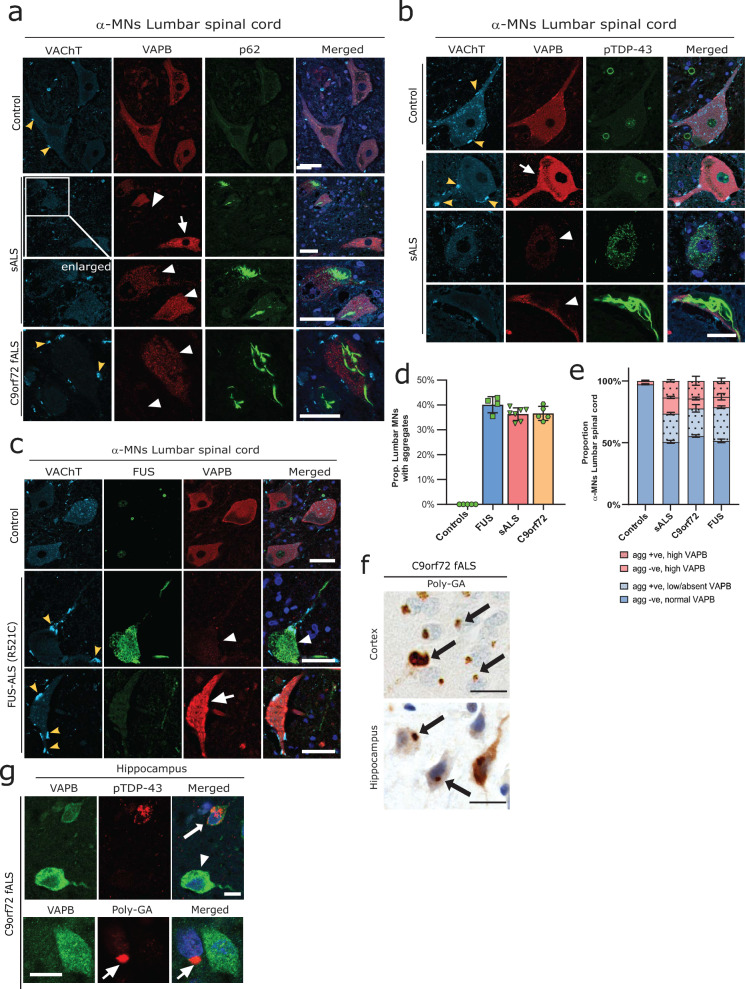
**a** Double immunofluorescence labeling of control, sALS and C9orf72 fALS (lower panel) lumbar spinal cord α-MNs using VAPB and p62 antibodies. We used VAChT antibody immunoreactivity (cyan, yellow arrowheads) to label α-MNs. The results show differential expression of VAPB in the presence (white arrowheads) and absence (white arrows) of p62 aggregates in sALS lumbar spinal cord α-MNs. Scale bars: 50 µm. Representative images from one of the three sections from sALS patients (n = 7) and age-matched normal individuals (n = 4). **b** Double immunofluorescence labeling of control and sALS lumbar spinal cord α-MNs using VAPB and pTDP-43 antibodies. The results show differential expression of VAPB in the presence (white arrowheads) and absence of pTDP-43 aggregates (white arrow) in sALS lumbar spinal cord α-MNs. Scale bar: 50 µm. Representative images from one of the three sections from sALS patients (n = 7) and age-matched normal individuals (n = 4). **c** Double immunofluorescence labeling was performed on control and FUS-ALS lumbar spinal cord α-MNs using VAPB and FUS antibodies. The results show reduced levels of VAPB in the presence (arrowheads) of FUS aggregates, while increased VAPB in the α-MNs (white arrows) were associated with absence of FUS aggregates. 3 sections from each patient and controls were analyzed. FUS-ALS patients (n = 3) and age-matched normal individuals (n = 3). Scale bars: 50 µm. **d-e** Quantification showing the percentage of VAPB immunoreactive (normal, low, or high immunoreactivity) α-MNs either harbouring (agg + ve) or not harbouring (agg -ve) pTDP43 aggregates in C9orf72 fALS and sALS cases and FUS aggregates in FUS cases. **d** Quantification showing the percentage of lumbar α-MNs in the presence or absence of cytoplasmic FUS in FUS-ALS and TDP-43 aggregates in sALS and C9orf72-fALS, respectively. **e** Antibody immunoreactivity on MNs was verified twice using one section per case each time. After confirming consistency, immunolabeling was performed in three sections per case. VAPB-immunoreactive MNs (low, medium, or high) with pTDP43 (C9orf72, sALS) or FUS aggregates (FUS ALS) were analyzed. Random sections were selected to avoid duplicate counts (Fig. 1 d-e). VAChT-positive, large α-MNs (> 50 µm) were manually counted using 20X and 40X objectives in the lumbar ventral horn. Three sections each from sALS (n = 7, α-MNs = 233), FUS ALS (n = 4, α-MNs = 137) and C9orf72 fALS (n = 5, α-MNs = 179) cases and controls (n = 4, α-MNs = 397) were analyzed. Agg: aggregates. **f** Detection of DPR (poly-GA) aggregates (arrows) in hippocampal as well as cortical neurons of C9orf72-fALS cases using DAB IHC. Scale bars: 25 µm. **g** Co-immunofluorescence labeling using VAPB, pTDP-43, and poly-GA antibodies showed differential expression of VAPB in the presence (white arrows) and absence (white arrowheads) of pTDP-43 aggregates (upper panel) and poly-GA aggregates neurons (lower panel) in hippocampal neurons of C9orf72 fALS patients. Representative images from one of the three sections from each panel (VAPB-pTDP-43 and VAPB-GA) C9orf72 fALS (n = 5). Scale bars: 30 µm

*Quantification (Human ventral horn alpha-MNs) *The antibody’s immunoreactivity on lumbar spinal cord ventral horn MNs was verified twice using one section per case each time. After confirming consistency, a final immunolabeling analysis was conducted in three non-adjacent sections per case. The semi-quantitative analysis of VAPB immunoreactivity (i.e. low, or high intensity of immunofluorescent staining) was determined in MN profiles containing pathological phosphorylated TAR-DNA binding protein (pTDP-43) aggregates (C9orf72 fALS and sALS) or FUS aggregates (FUS cases)., VAPB signal intensity was quantified using identical acquisition settings across all comparable samples, and thresholds for categorizing neurons as *high*, *normal*, or *low* VAPB immunoreactivity were established based on relative fluorescence intensity distributions normalized to internal neuronal populations within each section. The same threshold criteria were then consistently applied across all cases and experimental groups. To avoid inadvertent double counting of MN profiles, random sections were selected. For quantification, VAChT-positive large (> 50 µm) α-MNs with clear morphology in the ventral horn of the lumbar spinal cord (e.g. Figures 1g, h) were manually counted using 20X and 40X objectives. Three sections from each sALS patient (n = 7, total α-MNs = 233), *FUS* familial ALS patients (n = 4, total α-MNs = 137), *C9orf72 fALS* familial ALS patients (n = 5, total α-MNs = 179) and age-matched controls (n = 4, total α-MNs = 397) were analyzed.

#### Ethical approval

All procedures involving the use of post-mortem tissue samples were performed according to the ethical standards of the institutional and national research committees and the 1964 Helsinki Declaration and its later amendments. The studies were approved by the Ethical Committees of the Academic Medical Center, Amsterdam (W11_073). The postmortem tissues had been obtained within 6–30 h after death.

*Institutional Review Board Statement for the generation and use of the (human induced pluripotent stem cells) hIPSC lines* The performed procedures followed the Declaration of Helsinki (WMA, 1964) and were approved by the Ethical Committee of the Technische Universität Dresden, Germany (EK 393122012 and EK 45022009) and Rostock University of Technology, Germany (A 2019–0134). All patients gave written consent before any study-related analysis.

#### Cell culture, transient transfection, and treatments

##### Cell culture and treatment

Human epithelial cancer cells (HeLa) and human embryonic kidney cell line (HEK 293) cells were cultured in Dulbecco’s modified Eagle’s medium (DMEM, Invitrogen, Carlsbad, CA, USA), supplemented with 10% Foetal bovine serum (FBS) and 1% antibiotic/anti-mycotic solution (Invitrogen). Enhanced green fluorescence (EGFP)-P525L as well as Wt- Fused in sarcoma (FUS) stable HeLa cell lines, were kind gifts from Dr. Anthony Hyman through Dr. A. Hermann [74]. HeLa FUS-stable cell lines and national institute of health (NIH)—3T3 cells stably expressing EGFP- Microtubule-associated protein 1A/1B light chain 3 (LC3) were cultured in DMEM supplemented with 10% FBS, 1% penicillin/streptomycin, and puromycin (Sigma Aldrich). Cells were maintained in a humidified incubator at 37 °C and 5% CO_2_. Generation of NIH-3T3 cells stably expressing EGFP-LC3 or tandem mCherry-EGFP-LC3 with retroviral infection is described elsewhere [111].

#### Human iPSC-derived motor neurons (iPSC MNs)

Fibroblast cell lines were established from skin biopsies obtained from familial ALS patients and healthy controls [73]. The generation and characterization of control iPSC lines were reported previously [94]. Fibroblast lines were reprogrammed as previously described [57].

iPSC lines from human hair keratinocytes were generated as described in refs [40, 56] by a lentivirus containing a polycistronic expression cassette encoding for Oct4, Sox2, Klf4, and c-Myc [100] produced in 70% confluent 10 cm dishes with Lenti-X 293 T cells (Clontech, Mountain View, CA) by cotransfection of the polycistronic vector (8 mg), the pMD2 vector (2 mg), and the psPAX2 (5.5 mg) vectors (Addgene, Cambridge, MA) using 100 mL of the PolyFect transfection reagent (Qiagen, Hilden, Germany; www.qiagen.com). FUS iPSC and control cell lines were recently karyotyped using the HumanCytoSNP-12v array. All clones showing pathological SNPs were excluded. The generation of human neural precursor cells (NPCs) and MNs was accomplished following the protocol from Reinhardt and colleagues [93]. Briefly, the iPSC colonies were collected and stem cell medium containing 10 µM SB-431542, 1 µM dorsomorphin, 3 µM CHIR 99021, and 0.5 µM SAG (Cayman; 11,914) were added. After two days, the hiPSC medium was replaced with N2B27, consisting of the aforementioned factors, as well as DMEM/F12 and Neurobasal at a ratio of 50:50, with the addition of 1:200 N2 supplement, 1:100 B27 without vitamin A, and 1% penicillin, streptomycin and glutamine. On day 4, 150 µM ascorbic acid was added, and dorsomorphin and SB-431542 were withdrawn. Two days later, the EBs were mechanically separated and replated onto Matrigel-coated dishes. To this end, Matrigel was diluted (1:100) in DMEM-F12 and left on the dishes overnight at room temperature. The resulting small molecule NPCs (smNPCs) formed homogenous colonies during further cultivation. They were split at a ratio of 1:10–1:20 once a week using Accutase for 10 min at 37 °C and were not used beyond 10 consecutive passages [26]. For MN differentiation, we first derived NPCs which were maintained and differentiated into MNs as shown previously [74]. In brief, NPCs were maintained in basic medium (DMEM-F12/Neurobasal 50:50 medium, N2 supplement (1:200), B27 supplement without vitamin A (1:100), penicillin/streptomycin (1%), GlutaMAX (1%)), supplemented with Chiron 99,021 (3 μM), ascorbic acid (150 μM) and purmorphamine (0.5 μM) on tissue culture dishes coated with Matrigel. To induce the differentiation into MNs, NPC was split on the Matrigel-coated dish in the basic medium supplemented with BDNF (1 ng/ml), ascorbic acid (200 μM), retinoic acid (1 μM), GDNF (1 ng/ml) and purmorphamine (0.5 μM) and maintained for 5 days. For the final maturation, the medium was changed on day 6 to the basic medium supplemented with DBcAMP (100 μM), BDNF (2 ng/ml), ascorbic acid (200 μM), TGFβ-3 (1 ng/ml) and GDNF (2 ng/ml). Between days 7 and 10, the cells were split onto the dishes coated with poly-L-ornithine and laminin and maintained for at least 4–5 weeks before they were used for the final analysis. The cells were regularly tested for mycoplasma contamination.

#### Transient transfections

Cells were transfected to express an EGFP, hemagglutinin (HA), or mCherry tagged wild-type VAPB or with control EGFP, HA, or mCherry empty vectors. A detailed description of the generation of these plasmids is given elsewhere [6, 7, 72]. Transfection in the cell lines (HeLa, HEK293, NIH-3T3) was performed using Lipofectamine 2000 reagent (Invitrogen) according to the manufacturer’s recommendations, after 4 h incubation at 37 °C and 5% CO_2_ the transfection reagent-containing medium was replaced with fresh medium, and analysis was performed 48 h later.

#### Immunocytochemistry

HeLa, HEK293, and NIH-3T3 cells were cultured on µ-dishes (ibidi, GmbH) and transiently transfected to express either EGFP, HA or mCherry tagged wild type (Wt)—VAPB or to express control EGFP, HA or mCherry by using empty vectors. After 48 h cells were fixed in 4% PFA and processed for confocal microscopy. Permeabilization with 0.5% Triton X100 and blocking with 4% skimmed milk or normal goat serum was followed by primary antibody incubation overnight at 4 °C. Secondary Alexa488- or Alexa594-conjugated anti-mouse or anti-rabbit antibodies (Invitrogen) were used for visualization. Nuclei were stained with Hoechst 33,342 (1 µg/ml) or were mounted with DAPI containing fluorescent mounting media (DAKO) and visualized using a Zeiss LSM 700 confocal microscope (Zeiss, Oberkochen, Germany). Images were processed using the Zeiss LSM software and Adobe Photoshop CS5.

#### Filter trap assay (FTA) for detecting protein aggregates

Frozen autopsy tissues from lumbar spinal cord (control; n = 3 sALS; n = 6) and frontal cortex (control; n = 3 C9orf72; n = 5) were weighed (~ 60–80 mg/sample) and homogenized using a Dounce homogenizer in Triton lysis buffer (50 mM Tris–HCl, pH8.0, 150 mM NaCl, 1% Triton X-100 and 1% SDS in PBS) containing protease inhibitor cocktail (Roche Life Science, Penzberg, Germany) and incubated on ice for 30 min and followed by sonication for 3 s pulse at an amplitude of 30% using a Fisherbrand sonicator 100 sonic dismembrator.

For the cell culture experiments, cell pellets were collected after the experiments (3 independent experiments) and resuspended in Triton-X100-containing lysis buffer and processed identically like tissue samples. The crude lysates (both from the cell culture and tissue) were centrifuged for 10 min at 3000xg to obtain clear lysates (supernatants) and quantified for total proteins by the bicinchoninic acid (BCA) protein assay according to the manufacturer’s protocol (Thermo Scientific). A total of 100 µL of the supernatants were filtered through a 0.45 µm cellulose acetate membrane (OE 67, Whatman) using a 48-slot blot manifold (PR648, GE Healthcare). Before filtration, the membranes were immersed in Millipore H_2_O. SDS-resistant protein aggregates trapped by the filter were detected by immunoblotting described below. The FTA assay using the crude lysates (both from the cell culture and tissue) followed by WB analysis was performed at least 3 times.

#### Immunoblot analysis

Cells were scraped off the culture plate and centrifuged at 6000xg for 5 min to obtain cell pellets which were re-suspended in Triton X lysis buffer (50 mM Tris–Cl, pH8.0, 150 mM NaCl, 1% Triton X-100 in PBS, 0.5 mM PMSF and complete protease inhibitor mixture, Roche Applied Sciences) and with an amplitude of 8% for 10 s as described above. For immunoblot analysis performed on post-mortem tissue, frozen lumbar spinal cord tissue (control; n = 5, sALS; n = 9, C9orf72; n = 8) was weighed (~ 60–80 mg/sample), resuspended in Triton X lysis buffer (see above), and incubated on ice for 30 min followed by sonication as mentioned above. Clear lysates were obtained after centrifugation for 5 min at 2500 × g and protein concentrations were determined using the BCA method (Molecular Probes). Equal amounts of protein were boiled for 5 min in 1X SDS sample buffer (Bio-Rad protocol, https://www.bio-rad.com/webroot/web/pdf/lsr/literature/10007296D.pdf) and subjected to 10 SDS-PAGE electrophoresis at 20 mA/gel before being transferred to a polyvinylidene difluoride (PVDF) membrane, which had to be activated in methanol before use. Transfer lasted 1 h and 30 min at 350 mA and was followed by blocking in 4% skimmed milk in 0.08% Tween 20/Tris-buffered saline (TBS-T) for 30 min before incubation with primary antibody (Biorad protocol https://www.bio-rad.com/webroot/web/pdf/lsr/literature/10007296D.pdf). The dilutions for primary antibodies are described in Supplementary Table S1. After incubating the primary antibody overnight at 4 °C under gently shaking, membranes were washed three times with TBS-T for 10 min each and incubated with the appropriate horseradish peroxidase-conjugated secondary antibody for 1 h (antibody dilution 1:10,000) followed by the same washing procedure. Immunoreactive proteins were detected by enhanced chemiluminescence (Amersham Biosciences). Densitometric quantification of the band intensity was normalized to tubulin levels using Adobe Photoshop CS5.

#### Preparation of synaptic fractions

An enriched fraction of synaptic proteins can be obtained from isolated nerve terminals (i.e., synaptosomes). Synaptosomes contain the complete presynaptic terminal, including mitochondria and synaptic vesicles, with the postsynaptic membrane and the postsynaptic density. We used Syn-PER Synaptic Protein Isolation Reagent (Thermo Scientific, 87,793) and followed the protocol mentioned to effectively isolate functional synaptosomes containing active synaptic proteins. Briefly, frozen post-mortem lumbar spinal cord tissue (n = 3 control, ~ 50 mg) was homogenized in 10 volumes of the Syn-PER Reagent including protease inhibitors (Thermo Scientific, 87,785) using a 7 mL Dounce tissue grinder with 10 up-and-down even strokes. The homogenate was centrifuged at 1200 × g for 10 min to remove cell debris, and the supernatant (cytosolic fraction) was centrifuged at 15,000 × g for 20 min. The pellets (synaptic fraction), containing synaptosomes, were gently resuspended in the respective reagent and further proceeded for WB analysis.

*Quantification (VAPB at C-boutons)* VAChT-positive large (> 50 µm) α-MNs with clear morphology in the ventral horn of the lumbar spinal cord were manually counted using 20X and 40X objectives. C-bouton dimensions were measured using standardized image analysis under identical acquisition settings, and their number were manually counted. VAPB fluorescence intensity was quantified within defined synaptic regions of interest. Our analysis confirms that C-boutons in ALS MNs are significantly enlarged compared with controls, consistent with previous reports of synaptic remodeling in diseased motor neurons. In parallel, VAPB signal intensity at these synaptic structures shows altered distribution in ALS cases.

*Quantification (LC3 punctae)* For quantification of LC3 puncta in NIH3T3 GFP-LC3 cells **(**Fig. 7e**),** images were taken from random fields of the immunofluorescence-stained coverslips, and at least 30 cells were analyzed for large and small LC3 puncta. The criteria for large puncta were dependent upon brightness and relatively larger shape (Fig. 7d arrows), large (~ 0.7 µm and above) and small puncta (below 0.7 µm) with relatively less brightness intensity were considered as smaller puncta (white arrowheads). See Comparison. GraphPad Prism software and data were presented as bar graphs showing mean values ± SD.

*Quantification (cell culture, MNs)* Quantification of staining intensity of protein of interest within cells or MNs were performed by measuring the cytoplasmic staining intensity of region of interest of at least 40–50 MN from each group, or 10–20 cells/group, using Adobe Photoshop. Statistical analyses were done using GraphPad Prism software. Student’s t-test for comparison between two groups. * = *p*-value lower than 0.05; ** = *p*-value lower than 0.01. Values were expressed as mean ± SD. A.U = Arbitrary units).

#### Image acquisition

Images of the DAB-stained sections were taken with a Zeiss Axioplan microscope equipped with a 40 × objective and an Axio Cam 506 color camera (Zeiss). The exposure time and other imaging parameters were kept constant within each experimental set. Images from immunofluorescence labeled sections were taken with a Zeiss LSM 700 laser scanning confocal microscope using 20X, 40X, and 63X objectives. Images were acquired by averaging 4 scans per area of interest resulting in an image size of 1024 × 1024 pixels. The laser intensity and camera digital gain, exposure time were kept constant within the experiments for all the samples examined. Captured confocal images were analyzed using Adobe Photoshop CS5 and ZEN (Blue edition) 2009 software.

#### Statistical analysis

Statistical analyses were done using GraphPad Prism software (Graphpad Software Inc., San Diego, CA). For comparison between two groups, an unpaired and two-tailed Student’s t-test was used. Values in the graphs are represented as mean values ± SD. * = *p*-value lower than 0.05; ** = *p*-value lower than 0.01; *** = *p*-value lower than 0.001; **** = *p*-value lower than 0.0001. ns = not significant.

## Results

### Increased immunoreactivity of PQC factor VAPB inversely correlates with the absence of pathogenic aggregates in the MNs of multiple ALS subtypes

Endoplasmic reticulum (ER) chaperones play critical roles in regulating proteotoxic effects and autophagy overload due to the accumulation of misfolded protein aggregates in ALS [38]. Consistent with the role of ER chaperones in providing resilience against toxic proteins [14, 15, 21], we recently demonstrated that elevated levels of VAPB were found in AD transgenic (Tg) mouse models including APP/PS1 and pR5 Tau (Tg) mice [113]. Similarly, AD patients’ neurons affected by pathological phosphorylated tau (pTau) and granulovacuolar degeneration (GVD) showed differential immunoreactivity of VAPB, supporting the assumption that VAPB plays a role in maintaining neuronal PQC [113]. Thus, we hypothesized that VAPB supports selective neuronal survival by enhancing the autophagic clearance of toxic aggregates and that abnormal VAPB accumulation disrupts these protective mechanisms, contributing to neuronal vulnerability in ALS.

We focused on large diameter MNs in the lumbar spinal cord ventral horn as (putative) α-MNs which were recognized by their size (50–100 µm) as well as markers including vesicular acetylcholine transporter (vAChT) [23]. We confirmed the presence of VAPB immunoreactivity in these large VAChT-positive putative α-MNs (yellow arrowheads) in human lumbar spinal cord (Fig. 1a, b, c). These α-MNs frequently harbour pathologically phosphorylated TDP-43 (pTDP-43) and p62 aggregates in sporadic (s), as well as of C9orf72 familial (f) ALS (Fig. 1a, b, quantification d, e, Figure S1a,). In FUS fALS, α-MNs are pTDP-43-negative and FUS aggregate-positive (Figure S1b).

Consistent with our hypothesis and along with our previous observation on TDP-43 proteinopathies and the role of ER chaperones[43, 86, 108], we found increased VAPB immunoreactivity associated with the ER in human ALS lumbar spinal cord α-MNs (Fig. 1, Figure S1c) as well as in cortical neurons (Figure S3a) compared to the controls. α-MNs displaying increased immunoreactivity for VAPB were often devoid of p62 and pTDP-43 aggregates both in sALS as well as in C9orf72 fALS cases (Fig. 1a and b, arrows, Figure S1c). In contrast, surviving α-MNs already harboring p62 and/or pTDP-43 aggregates displayed significantly reduced immunoreactivity of VAPB (Fig. 1a, b, Figure S1c, arrowheads, quantification: Fig. 1d–e). Furthermore, we tested the pattern of VAPB immunoreactivity in lumbar spinal cord sections of FUS fALS patients. Again, α-MNs showing increased VAPB immunoreactivity were often devoid of FUS aggregates (Fig. 1c). In contrast, surviving MNs harboring FUS aggregates displayed significantly reduced levels of VAPB (Fig. 1c, Figure S1c, arrowhead, quantification d, e).

Dipeptide repeat (DPR) aggregates including poly-GA and poly-GR, together with pTDP-43 aggregates were abundant in cortical and hippocampal neurons and are central to the pathogenesis in C9orf72 fALS patients’ brains [32, 59] (Fig. 1f). In line with the VAPB immunoreactivity in α-MNs, we found similar differential immunoreactivity, where increased levels of VAPB negatively correlated with the absence of pTDP-43 and poly-GA aggregates, for example in C9orf72 fALS hippocampal neurons (Fig. 1g).

Immunoblot analysis performed on the lumbar spinal cord lysates obtained from sALS and familial C9orf72 fALS patients showed a clear decrease in VAPB protein levels (Figure S2 b-e). Together tthese results suggest that VAPB might exert selective neuroprotection by clearing pathogenic aggregates from the unaffected population of MNs in multiple subtypes of ALS.

In line with the results obtained above from the lumbar spinal cord, DAB immunohistochemistry performed on C9orf72- and FUS-ALS primary motor cortex showed differential cytoplasmic immunoreactivity, with many neurons showing reduced VAPB immunoreactivity (Figure S3a, white arrows) while a few neurons showed increased cytoplasmic immunoreactivity and VAPB accumulation (Figure S3a, black arrows, and red arrowhead respectively). In many instances, a peculiar, intense nuclear envelope immunoreactivity was also observed in several pyramidal neurons in the C9orf72 fALS and FUS-ALS motor cortex (Figure S3a, white arrowheads).

In parallel, similar patterns of VAPB immunoreactivity to those observed in human ALS were also found in the SOD1 mouse model of ALS. VAPB immunoreactive aggregates as well as ubiquitin-positive aggregates were also evident in the MNs of lumbar spinal cords of SOD1 mice (Figure S3c-d). Overall, VAPB protein levels were reduced, as detected by IF analysis, accompanied by increased proteotoxicity as evidenced by elevated ubiquitin accumulation (Figure S3d). Consistently, WB analysis revealed altered levels of the chaperones GRP78 and HSP70, along with increased ubiquitin levels, in the lumbar spinal cord of SOD1 mice (Figure S3e; quantification shown).

### ALS-resistant MNs display high levels of VAPB and are mostly devoid of pathogenic aggregates

**Fig. 2:**
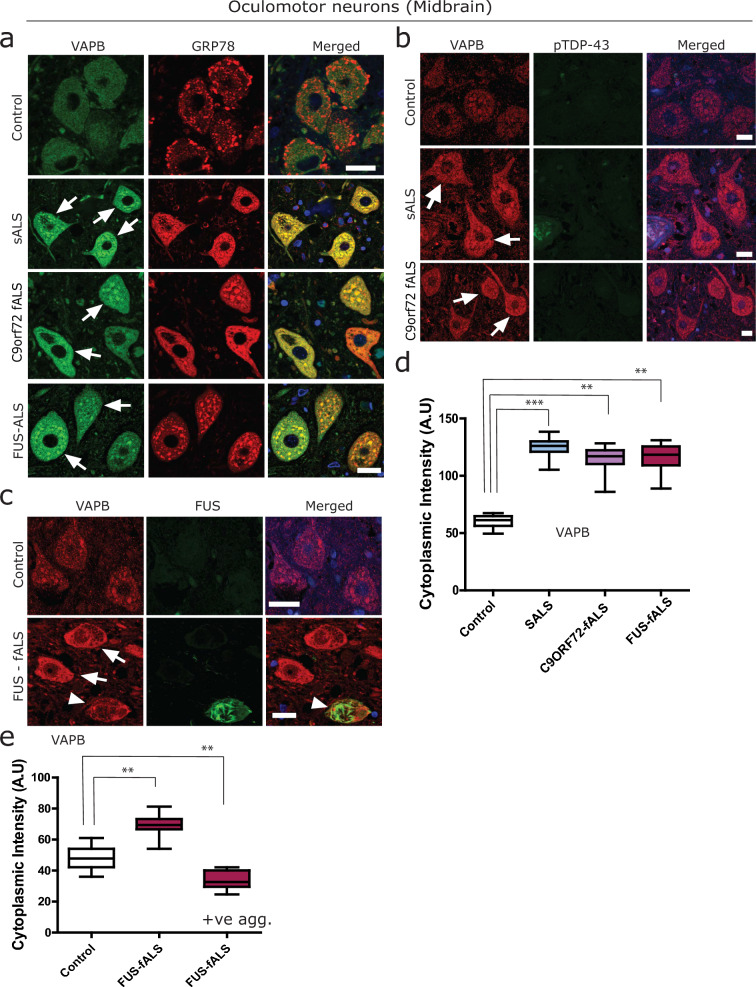
**a** Double immunofluorescence labeling performed on control as well as ALS midbrain oculomotor neurons using VAPB and GRP78 antibody showing significantly increased levels of VAPB as well as GRP78 (as a known ER stress marker, arrows) immunoreactivity in the oculomotor neurons of ALS subtypes compared to the controls. Three sections each were analyzed from control (n = 3), sALS (n = 3), C9orf72-fALS (n = 3), and FUS-ALS (n = 3) patients. Scale bars: 50 µm. **b** Double immunofluorescence labeling performed on control, sALS and C9orf72 fALS midbrain oculomotor neurons using VAPB and pTDP-43 antibodies showing significantly increased levels (arrows) of VAPB associated with the absence of pTDP-43 aggregates. Representative images from one of the three sections were each analyzed from control (n = 3), sALS (n = 3) and C9orf72-fALS (n = 3) patients. Scale bars: 50 µm. **(c)** Double immunofluorescence labelling performed on FUS-ALS midbrain oculomotor neurons using VAPB and FUS antibodies showed an overall increased level (arrows) of VAPB. Note the rare FUS aggregate bearing MN showing significantly reduced VAPB immunoreactivity (arrowhead). Representative images from one of three sections analyzed from FUS ALS patients (n = 3). Scale bars: 50 µm. **d, e** Quantification of the VAPB levels within the Oculomotor neurons were performed by measuring the cytoplasmic staining intensity of region of interest of at least 40–50 MNs from each groups, using Adobe Photoshop. Statistical analyses were done using GraphPad Prism software. Student’s t-test for comparison between two groups. * = *p*-value lower than 0.05; ** = *p*-value lower than 0.01. Values were expressed as mean ± SD. A.U = Arbitrary units)

As ALS progresses, specific sub-types of MNs preferentially deteriorate while others are spared until the disease’s end stage. For instance, MNs of Onuf’s nucleus in the sacral spinal cord and oculomotor nucleus in the midbrain exhibit resistance and are preserved in ALS [62, 81, 82]. Building upon this observation and our previous findings [43, 86]**,** we investigated whether these disease-resistant MNs also display increased immunoreactivity for VAPB. We chose to analyze midbrain oculomotor neurons (Figure S2a) because they are more easily accessible compared to MNs of the Onuf’s nucleus of the sacral spinal cord [62, 81, 82]. VAPB shows a Nissl-associated pattern of immunoreactivity in the MNs (Fig. 2) [43, 86, 108]. Consistent with the role of VAPB as a PQC factor, we observed a significantly elevated level of VAPB immunoreactivity in oculomotor neurons across various ALS sub-types when compared to controls (Fig. 2a, quantification d). For comparison, we utilized the known endoplasmic reticulum (ER) chaperone GRP78, a key player in the PQC mechanism that exerts neuroprotective effects by reducing levels of misfolded proteins [36]. As expected, we found increased levels of GRP78, which co-localized with VAPB (Fig. 2a).

We then investigated whether the elevated levels of VAPB correspond to reduced levels of aggregates in these MNs. Oculomotor neurons from both sALS and C9orf72 fALS appeared normal with no visible signs of atrophy or degeneration. They also exhibited increased levels of VAPB staining compared to control MNs. These MNs were largely devoid of any pathogenic pTDP-43 aggregates (Fig. 2b). In only a few cases we were able to observe occasional p62 or pTDP-43 immunoreactive profiles in these neurons (not shown). Similarly, FUS aggregates were also very rare in FUS-ALS oculomotor neurons (Fig. 2c, quantification e), and consistent with other sub-types of ALS, oculomotor neurons in FUS ALS displayed increased levels of VAPB. The high levels of VAPB in this population of MNs is consistent with the notion that it confers neuroprotection by preventing toxic aggregate formation.

### VAPB is sequestered with pathogenic aggregates in ALS: failure of PQC?

**Fig. 3:**
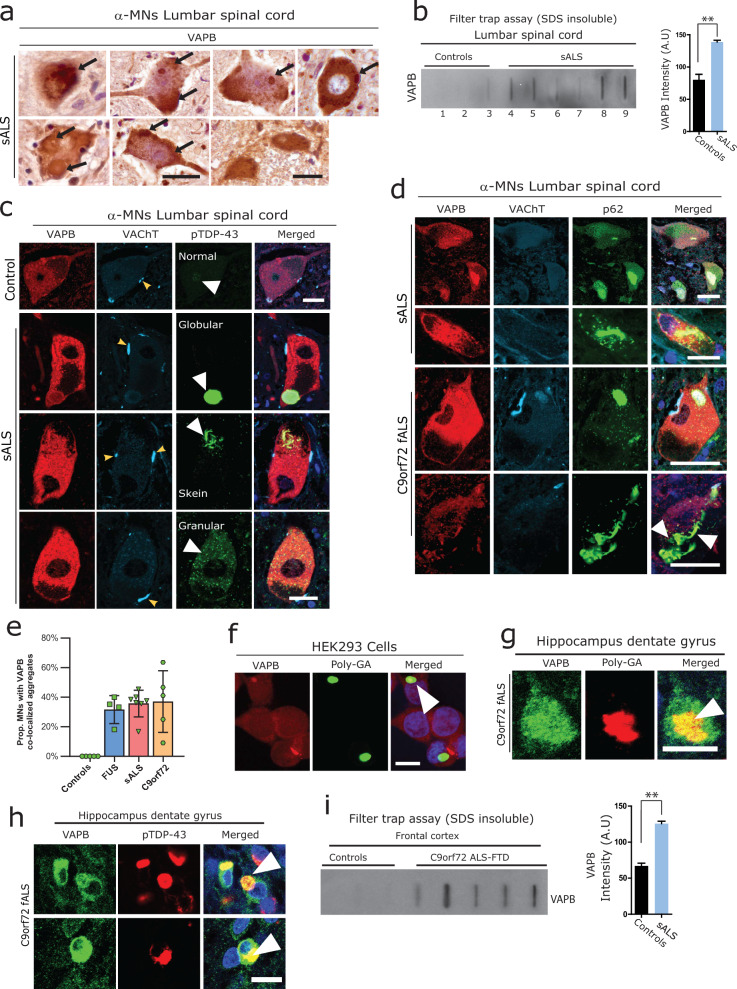
**a** DAB immunohistochemistry performed on sALS lumbar spinal cord α-MNs, showing accumulations of VAPB in various morphologies (arrows). Representative images from one of the 3 sections from sALS patients (n = 7). Scale bars: 50 µm. **b** Filter trap assay followed by Immunoblot analysis performed lysates obtained from control and sALS lumbar spinal cord showing SDS insoluble aggregates of VAPB in sALS. Controls, n = 3 (lane 1–3), sALS patients, n = 6 (lane 4–9). Corresponding densitometric data are shown, representing the relative band intensity of SDS insoluble aggregates. Statistical analyses were done using GraphPad Prism software. Student’s t-test for comparison between two groups * = *p*-value lower than 0.05; ** = p-value lower than 0.01. Values were expressed as mean ± standard deviation (SD) from three independent blots. A.U = Arbitrary units). **c–e** Double immunofluorescence labeling was performed on the lumbar spinal cord using VAPB antibody together with pTDP-43 antibody**(c)** or with p62 antibody in sALS cases and C9orf72-fALS cases. **d** Fluorescence showing co-localization of accumulated VAPB together with various morphologies of pTDP43 and p62 aggregates (arrowheads). Quantification showing % of VAChT positive α-MNs co-localized with the aggregates. **e** Three sections each were analyzed from sALS patients (n = 7), and C9orf72 fALS cases (n = 3). Scale bars: 50 µm. **f** VAPB immunolabelling performed on HEK293 cells overexpressing expanded poly GA plasmid showing the aggregation and sequestration of endogenous VAPB together with the aggregates of poly GA (red arrow). Scale bars: 10 µm. **g, h** Double immunofluorescence labelling was performed on hippocampal tissue using VAPB antibody together with either poly-GA (**g**) or pTDP-43 (**h**) antibodies in C9orf72 fALS cases, showing co-localization (red arrows) of accumulated VAPB and the respective aggregates. Semi quantitative analysis suggests that at least 26% of poly-GA aggregates and 20% of the pTDP-43 aggregates co-localized with VAPB (Table S3). Representative images from one of three sections analyzed from each C9orf72 fALS case (n = 3). (Scale bar: 20 µm. **i** A filter trap assay was performed using the same control, and C9orf72 fALS cases used for the immunoblot analysis previously (Figure S2 d), which revealed the presence of SDS insoluble aggregates of VAPB. Controls, n = 3 (lane 1–3) and C9orf72 fALS patients, n = 5 (lane 4–8). Corresponding densitometric data are shown on the right, representing the relative band intensity of SDS insoluble aggregates represented as FTA bands. Statistical analyses were performed using GraphPad Prism software. Student’s t-test for comparison between two groups. * = *p*-value lower than 0.05; ** = *p*-value lower than 0.01. Values were expressed as mean ± SD from three independent blots. A. U = Arbitrary units)

While the PQC mechanism typically maintains neuronal health during stress, protein misfolding, aggregation and persistent proteotoxic stress caused by an increased amount of misfolded protein aggregates can compromise the PQC mechanism [1, 34, 106]. This results in a decline in neuronal proteostasis and further contributes to neurodegeneration [34, 101, 106]. In line with this idea, we observed aggregated unfolded protein response (UPR) factor VAPB in the lumbar spinal cord α-MNs of human sALS (Fig. 3a). These aggregates exhibited various morphologies, reminiscent of the well-known pTDP-43 and p62-immunoreactive cytoplasmic neuronal aggregates in ALS (Figure S1a). To confirm the accumulated/aggregated VAPB, we performed the FTA with frozen lumbar spinal cord tissues from sALS and control patients. FTA analysis confirmed the presence of SDS-insoluble VAPB aggregates (Fig. 3b, quantification right). To gain further insights into the morphology of these SDS-insoluble VAPB aggregates in the α-MNs, we conducted co-immunolabelling experiments of VAPB along with pTDP-43 and p62, which are the known pathological hallmarks in the α-MNs of human sALS [75]. Consistent with earlier reports [8], we observed that pTDP-43 and p62 aggregates exhibited diverse morphologies, including dash-like, skein-like, and granular/globular-like forms (Figure S1), and were co-localized with accumulated VAPB (Fig. 3c, d; quantification in e) in the lumbar spinal cord α-MNs from sALS and C9orf72 fALS cases. Interestingly VAPB immunolabelling performed on HEK293 cells overexpressing the DPR (expanded poly GA) or mutant TDP43 showed the aggregation (arrow) and sequestration of endogenous VAPB together with the aggregates of poly GA and mutant TDP-43 (Figs. 3f and Figure S1d respectively). These findings were confirmed in affected cortical (not shown) and hippocampal neurons of C9orf72 fALS cases, where aggregated DPRs (poly-GA) and pTDP-43 co-localized with VAPB (Fig. 3g, h). Additionally, through biochemical analysis using FTA, we confirmed that VAPB forms SDS-insoluble aggregates in the lumbar spinal cord of C9orf72-fALS cases (Fig. 3i and quantification), like that observed in sALS cases. Furthermore, the presence of aggregated VAPB was in line with the observation that soluble levels of VAPB were significantly decreased in both sALS and C9orf72 fALS, lumbar spinal cord lysates, as detected by Western blot analysis (Figure S2b-e, quantification).

### VAPB is sequestered in FUS-ALS

**Fig. 4:**
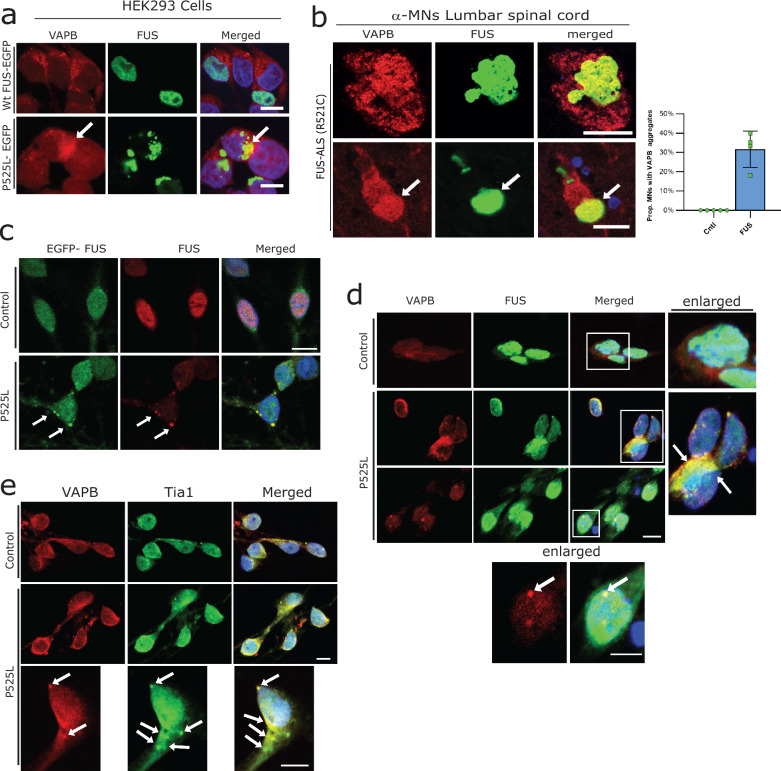
**a** VAPB immunolabelling performed on HEK293 cells overexpressing either the Wt FUS (upper panel) or the mutant FUS (lower panel) showing the aggregation (arrows) and sequestration of endogenous VAPB together with the aggregates of mutant FUS, see semiquantitative analysis table S3. Scale bars: 10 µm. **b** Double immunofluorescence labelling performed on FUS-ALS lumbar spinal cord α-MNs using VAPB and FUS antibodies showing co-localization of accumulated VAPB and FUS proteins. Representative images are shown from one of three sections from FUS ALS patients (n = 4). Scale bars: 50 µm. **c** Double immunofluorescence labelling of EGFP-FUS and FUS in P525L-FUS iPSC-derived MNs. Note the cytoplasmic FUS aggregates in P525L-FUS *iPSC*-derived MNs (white arrows),, see semiquantitative analysis table S3. Scale bars: 10 µm. **d** Double immunofluorescence labelling of EGFP FUS and VAPB in control and P525L-FUS—*iPSC* derived MNs. Note the sequestration of VAPB (white arrows) co-localizing with FUS aggregates in P525L-FUS MNs,see semiquantitative analysis table S3. Scale bars: 10 µm. **e** Double immunofluorescence labelling of VAPB and SG marker Tia1 in control and P525L-FUS iPSC-derived MNs (without EGFP- tag). P525L-FUS iPSC-derived MNs harbouring VAPB aggregates colocalize with Tia1 positive SGs (arrows), see semiquantitative analysis table S3. Scale bars: 10 µm

Our next objective was to analyze whether VAPB plays a similar role in FUS-ALS, particularly in cases with FUS-R521C or P525L mutations, which cause a rare, rapidly progressive and severe form of ALS. Interestingly, VAPB immunolabelling performed on HEK293 cells stably expressing mutant FUS showed the aggregation and sequestration of endogenous VAPB (see quantification (Table S3) together with the aggregates of mutant FUS (arrows, Fig. 4a). Consistent with this and with the previous observations (Fig. 3), lumbar spinal cord α-MNs from FUS-R521C cases also exhibited the accumulation of VAPB along with its sequestration with FUS aggregates (Fig. 4b, quantification right).

We then extended our investigation to FUS-ALS iPSC-derived MNs. These iPSC MNs have been demonstrated to faithfully represent FUS-ALS pathologies and exhibit age-dependent FUS aggregation (Fig. 4c [74, 105]). Immunolabelling using VAPB antibody showed significant aggregation of VAPB in FUS mutant iPSC-derived MNs, whereas iPSC-derived control MNs showed a normal distribution of VAPB (Fig. 4d). Interestingly, FUS aggregates were also found to be co-localized with VAPB aggregates (Fig. 4d, see semi quantitative analysis (Table S3), consistent with the VAPB sequestration with FUS aggregates observed in FUS-ALS lumbar spinal cord MNs.

Aggregates of RBPs, including FUS, often proceed via the stress granule (SG) pathway. FUS has also been reported as a component of SG [65, 85, 110]. In addition, T cell intracellular antigen-1 (TIA-1 or Tia1) is a prion-related RBPsR that is a well-known key component of SGs [60, 65]. In line with this, we also observed increased accumulations of Tia1-immunoreactive SGs (Fig. 4e). Intriguingly, these Tia1-positive SGs also sequester VAPB. See semi quantitative analysis (Table S3).

### VAPB is localized at the enlarged C-terminals of the lumbar spinal cord α-MNs in ALS

**Fig. 5:**
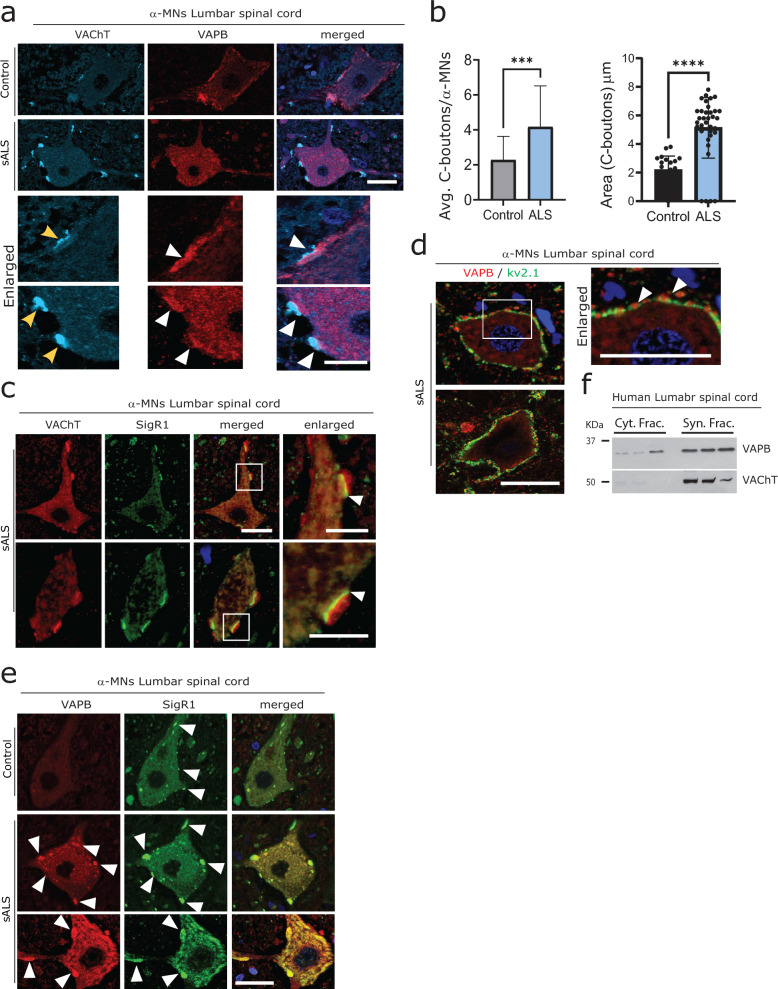
**a** Double immunofluorescence labeling performed on control as well as sALS lumbar spinal cord using VAPB (red) showing a peculiar C—bouton synapse (white arrowheads) marked by VAChT immunolabeling (cyan) and associated VAPB immunoreactivity in the α-MN of control and sALS lumbar spinal cord. Note the increased size of C—bouton synapse in sALS α-MN. Representative images from one of three sections were analyzed from sALS patients (n = 7) and age-matched normal control (n = 4). Scale bars: 50 µm. **b** Quantification showing MN count, number and enlarged area of C—bouton synapse. VAChT-positive large (> 50 µm) α-MNs with clear morphology in the ventral horn of the lumbar spinal cord were manually counted using 20X and 40X objectives. C-bouton dimensions were measured using standardized image analysis under identical acquisition settings, and their number were manually counted. (VAChT positive α-MN of control = 75 and sALS lumbar spinal cord = 22). **c** Double immunofluorescence labelling performed on the sALS lumbar spinal cord using SigR1 antibody together with VAChT antibody as a marker of presynaptic C-bouton synapse showing the postsynaptic localization of SigR1 (white arrowheads) juxtaposed to enlarged C-bouton synapse on the α-MNs. Representative images from one of three sections of the sALS patients (n = 3) were analyzed. Scale bars: 30 µm. **d** Double immunofluorescence labeling performed on control as well as sALS lumbar spinal cord using VAPB and SigR1 antibody showing their accumulation and sequestration at the C—bouton synapse (white arrowheads) in α-MNs of sALS lumbar spinal cord. Representative images from one of three sections from sALS patients (n = 7) and age-matched normal (n = 4). Scale bars: 50 µm. **e** Double immunofluorescence labelling performed on sALS lumbar spinal cord using VAPB antibody together with post-synaptic KV2.1 antibody showing the presence of VAPB at its vicinity. Representative images from one of three sections from ALS patients (n = 3). Scale bars: 50 µm. **f** Immunoblot analysis of the lysates obtained from purified cytosolic and synaptic fractions (Syn Frac, Cyto. Frac.) using VAPB and presynaptic marker VAChT antibody, showing the enrichment of VAPB at the synaptic fractions. n = 3 control spinal cord was used for making the lysates

While examining VAPB staining in lumbar spinal cord α-MNs, we noticed a distinct VAPB signal at a specific type of synapse called the C-bouton (Fig. 5a, white arrowheads). Earlier studies, including ours, have shown that C-bouton synapses become enlarged in ALS MNs, likely as a response to ongoing neuron loss. These synapses can be identified using VAChT staining (Fig. 5a, yellow arrowheads). Interestingly, in ALS patient lumbar spinal cord α-MNs, VAPB staining was also present at these enlarged C-bouton synapses (Fig. 5a, quantification b) [88]. The enlarged C-bouton synapse is associated with several pre- and post-synaptic signaling proteins, including the ER chaperone SigR1, which accumulates at the post-synaptic side (Fig. 5c, white arrowheads). Consistent with this, we observed that VAPB co-localized with SigR1 at these sites (Fig. 5d, white arrowheads) [86]. Furthermore, VAPB immunoreactivity at the synaptic sites was confirmed by the presence of VAPB in the vicinity of postsynaptic Kv2.1 (Fig. 5 e, white arrowheads). Finally, the presence of VAPB at the synaptic sites was confirmed using WB analysis from the synaptic fraction purified from the human lumbar spinal cord (Fig. 5f).

### Endogenous VAPB is a substrate for autophagy and the ubiquitin proteasome system

**Fig. 6:**
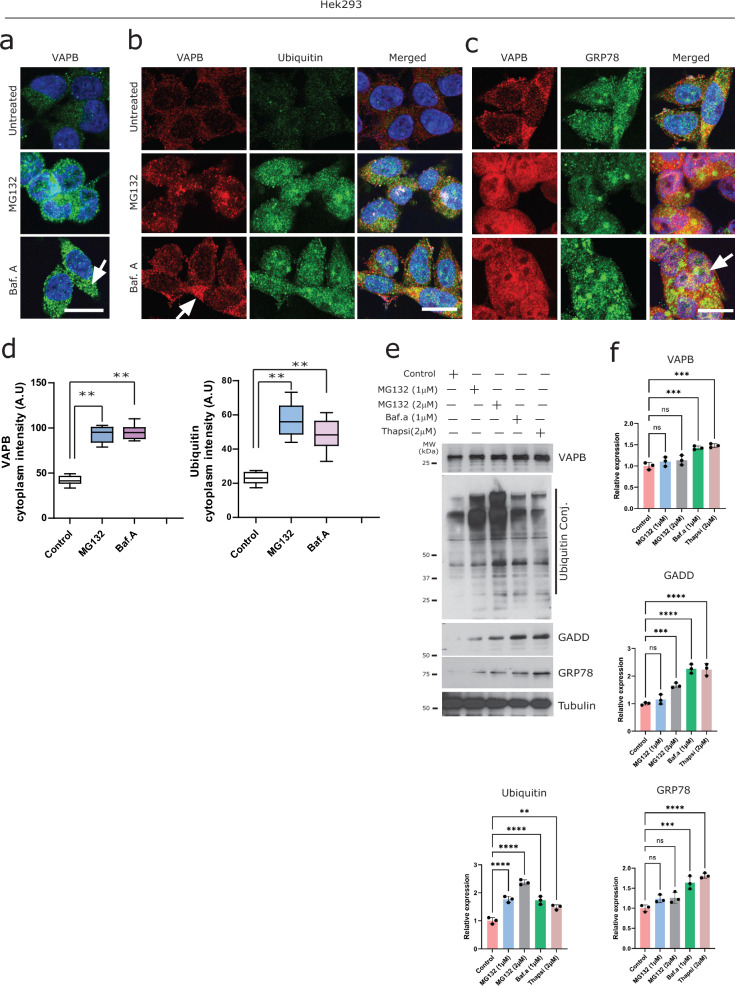
**a** VAPB immunofluorescence labeling was performed on HEK293 cells, which were treated with the autophagy inhibitor Bafilomycin A (Baf.A) or the proteasome inhibitor MG132. Note the accumulation of VAPB after Baf.A (arrows) as well as MG132 treatment; for quantification, see (**d**). Scale bar: 10 µm. **b-d** Double-immunofluorescence labeling using VAPB and Ubiquitin (**b**) as well as VAPB and ER stress marker GRP78 (**c**), performed on HEK293 cells treated with Baf. A or MG132. Scale bars: 10 µm. Note the accumulation of VAPB and GRP78, especially after Baf.A (arrows) compared to MG132 treatment. Quantification of VAPB intensity (**d**). Quantification was performed by measuring the cytoplasmic staining intensity of region of interest using Adobe Photoshop from at least 10 cells/treatment groups. Statistical analyses were done using GraphPad Prism software. Student’s t-test for comparison between two groups. * = *p*-value lower than 0.05; ** = *p*-value lower than 0.01. Values were expressed as mean ± SD. A.U = Arbitrary units). **e** Immunoblot analysis of NSC-34 cells treated with Baf. A, 1 and 2 µM, MG132, 1 and 2 µM, as well as ER stress marker and autophagy inhibitor Thapsigargin (2 µM) for 6 h. Note the accumulation of endogenous VAPB and GRP78, especially in ER stress and after Baf. A compared to MG132 treatment. **f** Quantification: Corresponding densitometric data were obtained from three independent experiments. The relative band intensity of VAPB, GADD, and GRP78 normalized with tubulin levels showing their increased levels under the treatment conditions. Statistical analyses were done using GraphPad Prism software. Student’s t-test for comparison between two groups ns = not significant; * = *p*-value lower than 0.05; ** = *p*-value lower than 0.01; *** = *p*-value lower than 0.001; **** = *p*-value lower than 0.0001. Values were expressed as mean ± SD from three independent blots. A. U = Arbitrary units)

VAPB was found to be aggregated in the ALS autopsy tissues and IPSC-derived MNs obtained from FUS-ALS patients. This phenomenon is intriguing because endogenous soluble VAPB levels are reduced in sALS and C9orf72 fALS lumbar spinal cord lysates (Figure S2 b-d) and FTA analysis in these samples showed increased SDS resistant insoluble VAPB aggregates (Fig. 3b and i). Previous studies have demonstrated that both loss and toxic gain of mutant VAPB functions are associated with neurodegeneration [44, 49, 104] with disturbed autophagy being central to the pathogenesis [27, 51]. We therefore investigated (a) whether inhibition of autophagy could lead to the accumulation of VAPB, or vice versa, and (b) whether an accumulation of VAPB in any given instance are due to the failure of autophagy. To confirm this hypothesis, we treated cells with a known autophagy inhibitor Bafilomycin A (Baf.A) or with a proteasome inhibitor MG132 and then checked the level of VAPB under these conditions. As expected, blocking autophagy led to the increased accumulation of VAPB in HEK293 cells compared to MG132 treated cells (Fig. 6a). Co-immunolabelling of VAPB with either ubiquitin antibody or the ER stress marker GRP78 confirmed the proteotoxicity and increased ER stress upon Baf.A treatment (Fig. 6b-d, quantification). Consistent with the findings, immunoblot analysis of HEK293 cells treated with either Baf.A or the ER stressor thapsigargin showed increased levels of VAPB. Besides, increased levels of ubiquitin and GRP78 and GADD-153 protein further confirmed the ongoing proteotoxic effect upon these inhibitors (Fig. 6 e, f, quantification). In summary, these results indicate that VAPB is a substrate of autophagy and its accumulation/aggregation in cells/neurons indicates disturbed autophagy.

### VAPB regulates autophagy: Increased turnover of autophagy substrates by controlled over-expression of VAPB

**Fig. 7:**
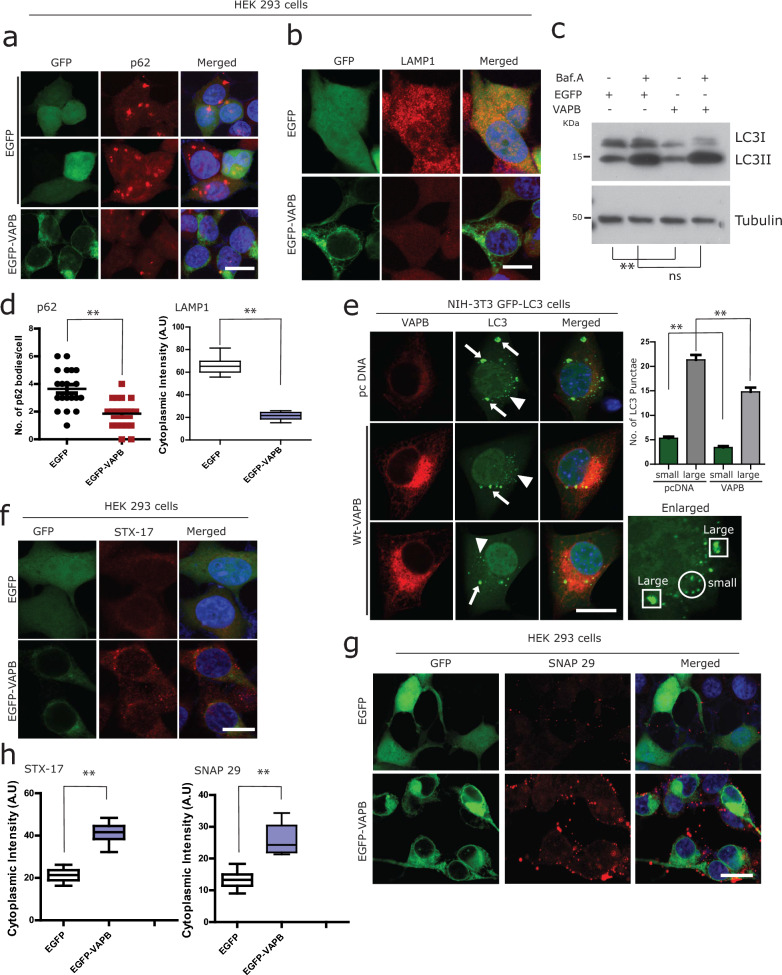
**a-b** Immunofluorescence labelling using p62 (**a**) and Lamp1 (**b**) antibodies showing decreased levels of p62-positive bodies as well as Lamp1 granular bodies (suggesting their clearance) in HEK293 cells overexpressing EGFP-VAPB (after 24 h) compared to control EGFP vector—scale bars: 10 µm. **c** Immunoblot analysis using LC3 antibody to analyze the autophagy flux in HEK293 cells overexpressing EGFP-VAPB or control EGFP vector treated with autophagy inhibitor Bafilomycin A (Baf. A, 2 µM for 6 h). Corresponding densitometric data are shown; representing the relative band intensity of LC3-II/LC3-I, normalized with tubulin levels. GraphPad Prism software. Student’s t-test for comparison between two groups. ns = insignificant; ** = *p*-value lower than 0.01; Values were expressed as mean ± SD from three independent blots. A. U = Arbitrary units). **d** Quantification of the number of p62 bodies as well as cytoplasmic intensity of LAMP1. For quantification p62 bodies, at least 20 GFP positive cells were counted for each condition (GFP-Control and EGFP-VAPB), number of p62 bodies were counted manually. Quantification for the LAMP1 levels was performed by measuring the cytoplasmic staining intensity of region of interest using Adobe Photoshop from at least 10 cells/groups. Statistical analyses were done using GraphPad Prism software. Student’s t-test for comparison between two groups. * = *p*-value lower than 0.05; ** = *p*-value lower than 0.01. Values were expressed as mean ± SD. A.U = Arbitrary units). **e** NIH-3T3 EGFP-LC3 cell lines stably expressing LC3 were transfected with merry-VAPB showing the clearance of LC3 large (~ 0.7 µm and above) and small punctae (below 0.7 µm) upon VAPB expression (Note the no. of large LC3 punctae, arrows) as well as the small punctae (arrowheads) are reduced upon VAPB overexpression. EGFP-LC3 punctae (small and large) were manually counted (see inset) for at least 30–40 cells. Quantification. Scale bars: 15 µm. **f-g** Immunofluorescence labeling using STX17 antibody (**f**) and SNAP29 antibody (**g**) showed increased levels of STX17 and clearance of SNAP29 protein levels in cells overexpressing VAPB-EGFP (after 24 h) plasmids (representing increased autophagy) compared to the overexpression of control EGFP vector. Scale bars: 10 µm. **h** Quantification for the STX17 and SNAP29 levels was performed by measuring the cytoplasmic staining intensity of region of interest using Adobe Photoshop from at least 10 cells/groups. Statistical analyses were done using GraphPad Prism software. Student’s t-test for comparison between two groups. * = *p*-value lower than 0.05; ** = *p*-value lower than 0.01. Values were expressed as mean ± SD. A.U = Arbitrary units)

The present study observed that VAPB interacts with the autophagy protein p62 (Fig. 3d) and that VAPB levels increase when autophagy is inhibited (Fig. 6) and we also proposed that VAPB confers reduced vulnerability to toxic aggregates across multiple ALS sub-types (Figs. 1 and 2). VAPB interacts with autophagy related (ATG) proteins to maintain endoplasmic reticulum (ER) and the isolation membrane (IM), ER/IM contacts which are essential for autophagosome biogenesis. However, considering the deleterious impact of the P56S-VAPB mutation on autophagosome biogenesis and late autophagy stages [108, 114], we hypothesize that increasing the levels of wild-type (Wt) VAPB can enhance autophagic flux. Supporting this hypothesis, we demonstrated that VAPB over-expression effectively degrades p62 bodies by inducing autophagy (Fig. 7a, quantification 7d). Additionally, we observed reduced LAMP1 immunoreactivity, indicating enhanced turnover of LAMP1 due to increased autophagy activity (Fig. 7b, quantification 7d). These results were consistent with our previous findings [43, 108] suggesting the role of VAPB in managing autophagy.

To further confirm that VAPB protein facilitated the induction of autophagy, we used Baf. A to block autophagy in cells overexpressing either control EGFP-, or EGFP-VAPB (Fig. 7c) or control mCherry or VAPB-mCherry and monitored the protein levels of LC3 as an indicator of autophagy flux [69, 97]. In line with previous findings, we observed a clear reduction in LC3II levels in cells overexpressing VAPB compared to EGFP-transfected control and a significant accumulation of LC3II in Baf.A treatment compared to the control (Fig. 7c). To further verify these results, we used a mouse fibroblast (NIH-3T3) cell line stably expressing EGFP-LC3 (see Materials and Methods), that was characterized by a baseline autophagic activity [70] showing both large and small LC3 punctae (Fig. 7e, enlarged panel). Upon VAPB overexpression, we noted a decrease in the number and size of these LC3 punctae, indicating clearance of LC3 vesicles through autophagy induction (Fig. 7e, enlarged panel and quantification) [69, 97]. Furthermore, increased levels of autophagosome-lysosome fusion proteins, including STX17 and SNAP29 [41] were representative of autophagy activation upon VAPB overexpression (Fig. 7f, g, quantification h).

### Overexpression of Wt-VAPB facilitates clearance of ALS-associated mutant RBP aggregates via autophagy

**Fig. 8:**
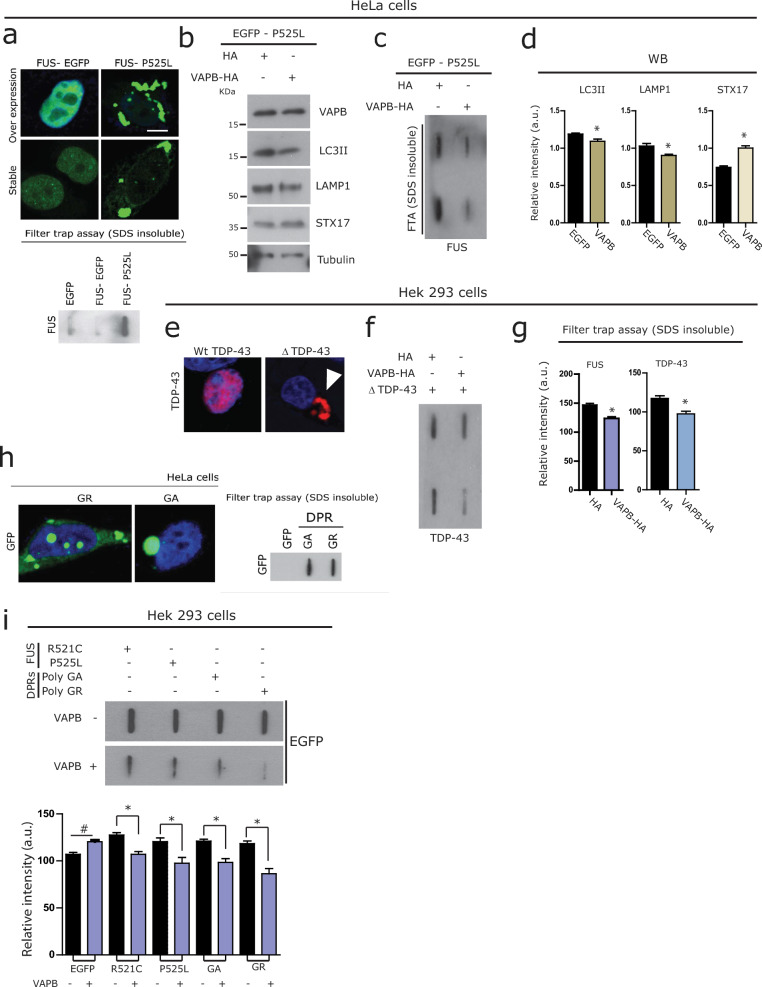
**a** Normal nuclear FUS localization of Wt FUS in HeLa cells either over expressing or stably expressing FUS-EGFP Wt construct (left panel) or cytoplasmic aggregation of mutant FUS when expressing mutant FUS-P525L-EGFP (right panel). FTA showing presence of SDS insoluble FUS aggregates in mutant FUS-P525L-EGFP expressing cells compared to the control FUS-EGFP Wt expressing cells. **b–d** HeLa cells stably expressing P525L FUS were transfected with HA-VAPB or a control vector. Cells were collected 24 h post-transfection; cell lysates were prepared for Western blot (WB) and Filter Trap Assay (FTA) separately to assess the autophagy flux using WB and FUS aggregation using FTA. Activation of autophagy was indicated by the reduced intensity of LC3II and LAMP11 bands in the Western blot analysis (**b**). Followed by the autophagy induction by VAPB, FTA showed a significant reduction of SDS-insoluble FUS aggregates in cells overexpressing VAPB (**c**). Quantification: Corresponding densitometric data were obtained from three independent experiments. The relative band intensity of LC3II, LAMP1, and STX17 normalized with tubulin levels (**d**). Statistical analyses were done using GraphPad Prism software. Student’s t-test for comparison between two groups; * = p-value lower than 0.05. **e–g** Hek293 cells showed normal nuclear localization of TDP-43 when transfected with Wt- TDP-43 plasmid and cytoplasmic aggregates of TDP-43 when transfected with deletion mutant (delta TDP43) plasmids (**e**). FTA was performed on the cells overexpressing HA-VAPB or a control vector together with delta TDP-43 truncated plasmid. Note the reduction of TDP43 aggregates in cells overexpressing HA-VAPB **(f)**. Corresponding densitometric data are presented, SDS-insoluble aggregates as FTA bands (**g**). Statistical analysis using GraphPad Prism software. Student’s t-test for comparison between two groups. ns = not significant; * = *p*-value lower than 0.05. Values were expressed as mean ± standard deviation (SD) from three independent blots. A. U = Arbitrary units). (**h**) HeLa cells showed aggregates of DPRs (GR, GA) when transfected either with expanded poly GA or poly GR plasmids (left panel). FTA (right panel) was performed on lysates obtained from transfected cells showing SDS insoluble aggregates of GA and GR. **(i)** FTA was performed on lysates obtained from HEK293 cells overexpressing DPRs (poly GA and poly GR) and mutant FUS (R521C and P525L), along with either HA-VAPB or control vectors. The assay showed a reduction in SDS-insoluble FUS, Poly-GA, and Poly-GR aggregates in cells overexpressing VAPB protein**.** Corresponding densitometric data are presented (lower panel), representing the relative band intensity of SDS-insoluble aggregates as FTA bands. Statistical analysis using GraphPad Prism software. Student’s t-test for comparison between two groups. ns = not significant; * = *p*-value lower than 0.05. Values were expressed as mean ± SD from three independent blots. A. U = Arbitrary units

Autophagy is the primary pathway for degrading misfolded proteins, and VAPB plays a crucial role in this process [108, 114]. Therefore, we hypothesized that VAPB could facilitate the degradation of pathogenic aggregates via activation of the autophagy pathway. To investigate this hypothesis, we utilized VAPB-induced autophagy to monitor the clearance of different types of ALS-associated toxic aggregates. We used mutant EGFP-P525L as well as Wt- FUS stable cell lines [74], in which P525L mutant FUS forms cytoplasmic aggregates (Fig. 8a), which are also SDS insoluble (Fig. 8a, lower panel). We expressed HA-VAPB or a control plasmid in these cell lines to assess autophagy flux and monitor FUS aggregation with or without VAPB. Western blot analysis of the cellular lysates confirmed the induction of autophagy by VAPB, evidenced by reduced levels of LAMP1 and LC3II (Fig. 8b, d: quantification). Consistent with activated autophagy, VAPB expression significantly reduced the SDS-insoluble aggregates of FUS (Fig. 8c, g: quantification). Using a similar approach on cell line overexpressing mutant (N-terminal deletion)—TDP43 (delta TDP-43) which forms cytoplasmic aggregate (Fig. 8e), that can be biochemically resolved by FTA as SDS-resistant aggregates (Fig. 8f). Overexpression of Wt-VAPB (HA-VAPB) reduced such TDP-43 aggregates (Fig. 8f, quantification g).

Encouraged by these findings, we performed a similar set of experiments with the overexpression of Wt-VAPB and other FUS-ALS mutants and C9orf72-associated dipeptide repeat (DPR) aggregates causing mutants. C9orf72-associated dipeptide repeat (DPR) aggregates causing mutants also forms cytoplasmic and nuclear aggregates, that can be detected by FTA as well (Fig. 8h). Consistent with earlier results, we observed a significant reduction in SDS-insoluble aggregation levels of these mutant proteins (Fig. 8i, quantification). These results confirm the role of VAPB in regulating autophagy and managing the neuronal PQC.

## Discussion

In this study, we investigate the neuroprotective effects of VAPB. Our findings demonstrate that neurons containing pathogenic aggregates exhibit reduced levels of soluble VAPB proteins. Conversely, neurons lacking aggregates display increased levels of VAPB. Disease-resistant oculomotor neurons exhibit elevated levels of VAPB proteins and are free from pathological aggregates. Consistent with this, overexpression of VAPB facilitates the degradation of pathological aggregates through autophagy. These results suggest a neuroprotective role for VAPB in ALS.

Little is known regarding why only specific MNs/neuron subtypes preferentially deteriorate, and other MNs/neurons such as MNs of the oculomotor and Onuf’s nucleus tend to be spared in ALS [61, 81, 82]. Moreover, in ALS model mice (SOD1G93A), high-firing-threshold fast fatigable (FF) MNs are most vulnerable (being prone to ER stress, and protein aggregation) compared to low-firing-threshold slow (S) MNs which express more protective factors[21, 98]. In addition, vulnerable neurons have a higher propensity to accumulate disease-related misfolded proteins, probably due to the lack of protective factors and biochemical features. [21, 98]**.** Consistent with the above notions, we observed that the affected neurons harbouring pathogenic aggregates, including pTDP-43 and FUS, showed reduced VAPB, while neurons displaying increased levels of VAPB were often devoid of such aggregates. In addition, we also showed that VAPB was often found to be sequestered within these toxic aggregates, and the levels of soluble VAPB proteins were reduced. Reduced VAPB levels in ALS MNs as well as in IPSC MNs were reported previously [5, 68, 107]. The present findings have further strengthened our hypothesis, showing that ALS-resistant midbrain oculomotor neurons were equipped with high levels of VAPB proteins and were devoid of pathological p62 and TDP-43 aggregates. These data suggest a neuroprotective role conferred by VAPB, where MNs/neurons with higher levels of VAPB are spared from degeneration in the early- and mid-stages of ALS.

The presence of selective degeneration of pTDP-43 inclusion-bearing neurons supports the notion that pTDP-43 aggregates are tightly linked with neurodegeneration [52]. Other groups have observed that the extent of pTDP-43 pathology correlates with neuronal loss across different regions of the CNS [76, 91, 92]. The frequently observed presence of dash-like pTDP-43 immunoreactive inclusions may represent an early stage of pTDP-43 accumulation [8, 76], and neuronal death may occur only with further aggregation, leading to massive deposits encompassing large portions of the neuronal cell body and its neurites. Our observation of VAPB immunoreactivity alongside pTDP-43 and FUS lesions suggests that misfolded protein aggregates may trigger a self-perpetuating cascade that propagates pathological assemblies. The sequestration of VAPB within these inclusions implies a modulatory role in this process and may reflect several non-mutually exclusive possibilities, including a failed protective response, attempted participation in aggregate processing, or passive entrapment secondary to ER disruption and proteostasis collapse. Together, these findings indicate that VAPB acts in a chaperone-like capacity to constrain pathological phase transitions, preserve proteostasis, and sequester misfolded proteins. The selective expression of VAPB in ALS α-MNs and its co-localization with aggregates further underscore its neuroprotective function within protein quality control networks [8, 64, 76].

These misfolded proteins may be redirected for refolding or be targeted for degradation if refolding to their usual native structure is unsuccessful. The proteins and chaperones directing the process, such as VAPB, often become permanently sequestered with the pathogenic aggregates, exerting their toxic gain-of-function or loss-of-function effects [13, 34, 47, 96]. These results are consistent with previous findings where proteins functioning similarly to VAPB, such as other ER chaperones (e.g. SigR1, SIL1/BiP complex, heat shock proteins (Hsp) including Hsp70, Hsp40, and Hsp60) safeguard other proteins against stress-induced misfolding and aggregation [33]. Here in this regard, it’s logical to place VAPB within the broader protein quality control network. VAPB may act in parallel with or converge functionally on other protective yet aggregate-associated PQC factors, including p62, OPTN, and VCP, which are likewise recruited to pathological inclusions while participating in cargo recognition, trafficking, and degradation. Thus, defining the relationship between VAPB and these PQC pathways will be important for understanding whether VAPB sequestration reflects a compensatory proteostatic response or a more terminal failure of clearance machinery.

Aggregation of ALS-associated RBPs proceeds through the SG pathway [3, 65], and RBPs, including SG components, are particularly susceptible to aggregation due to the presence of their RNA-binding- and prion-like domains, which contribute strongly to aggregate formation under stressful stimuli, including chronic autophagy impairment [90]. On a similar note, the findings of the current study are consistent with the above concept as well as with our previous findings of SG accumulation and autophagy impairment in VAPB ALS-8 patients’ muscle biopsies [43, 108].

Autophagy coupled with PQC processes clears toxic aggregates and other cellular waste. Autophagy is a tightly regulated, multi-step process orchestrated by several ATG-dependent and independent proteins [30, 80]. Apart from the ATG genes, recent reports have identified several ER-associated genes including VAPB, which regulate autophagy at multiple levels. VAPB interacts with ATG proteins, contributing to ER/IM contacts and promoting autophagosome biogenesis [114]. Based on our observation of selective neuronal vulnerability associated with VAPB, we hypothesized that VAPB facilitates this function by specifically targeting pathogenic aggregates and clearing them through the activation of autophagy (aggrephagy), similar to various other proteins [7, 50, 54, 114]. Previous studies have demonstrated that VAPB plays a critical role in regulating autophagy by modulating key proteins in the pathway [112]. Specifically, VAPB knockdown leads to the upregulation of Beclin 1, a central initiator of autophagy, promoting LC3 conversion and puncta formation —essential steps in autophagosome biogenesis. Conversely, increased VAPB levels have been shown to suppress autophagy-related processes, suggesting that reduced VAPB may enhance autophagy activity [113]. However, this study does not address the potential toxicity associated with VAPB loss-of-function, which has been reported by others. In contrast, Zhao et al. [114] proposed a crucial role for VAPB in autophagosome biogenesis and the initiation of autophagy, partly through interactions with ATG proteins. These studies investigate autophagy at distinct stages using different experimental systems and tools, indicating that VAPB likely regulates autophagy through multiple mechanisms. Our findings are partially consistent with this view, as we observe that VAPB influences both early and late stages of autophagy. This highlights the complexity of VAPB’s function and calls for future studies integrating detailed protein–protein interaction analyses with transcriptomic profiling. Building on this, we propose that VAPB plays a selective and context-dependent role in neuronal vulnerability by facilitating the autophagic clearance of pathogenic protein aggregates (aggrephagy), thereby contributing to proteostasis and neurodegeneration. We provide evidence that VAPB overexpression enhances the degradation of TDP-43, FUS, and DPR aggregates through autophagy induction. While the precise spatiotemporal dynamics of VAPB-mediated aggrephagy remain to be fully elucidated, VAPB is known to recruit and stabilize ULK1 at FIP200 puncta, a key step in autophagosome formation at the ER [114]. We propose that VAPB promotes autophagosome biogenesis near aggregate formation sites, thereby enhancing their clearance.

It has become clear that mutations in genes regulating autophagy receptors and/or mutations in several ER proteins, including SigR1, SIL1, HSPB1, HSPB8, and HSJ1, cause defects in ER structure, which then impairs autophagy leading to familial neurodegenerative disorders, including MN diseases [2, 10, 11, 19, 48, 95, 99]. Furthermore, ER chaperones, including GRP78, SigR1, and SIL1, as well as ER tethering proteins such as VAPB, are determinants of ER functions, including PQC/UPR and autophagy [21, 35, 36, 71]. Moreover, these proteins are abnormally modified in neurodegenerative conditions such as AD [20, 39, 67, 109], PD [67], HD [66], and ALS [21, 29, 43], diseases that feature distinct ultrastructural ER alterations and defective protein degradation pathways [16, 49, 103]. Thus, our study suggests that strategies to enhance aggrephagy, including AAV-9-mediated viral overexpression of VAPB in vivo ALS models, could have a beneficial therapeutic effect.

Synaptic dysfunction and loss are hallmark features of neurodegenerative diseases, including ALS. VAPB, along with PTPIP51 ER-mitochondria tethers, localizes to synapses, where they interact to regulate various synaptic functions [28]. Notably, we identified a distinct focal sub-surface C-bouton associated with VAPB and SigR1 immunoreactivity in both normal and ALS patient α-MNs, with these immunoreactive zones significantly enlarged in ALS α-MNs. These findings align with our observations and those of Pullen et al. [88, 89], who reported an apparent increase in C-terminal size in ALS patients and G93A SOD1 mouse α-MNs during disease progression. In this context, the elevated VAPB immunoreactivity in C-terminal territories supports the concept of a compensatory neuroprotective response aimed at preserving VAPB’s functional role following MN loss. This idea is reinforced by studies showing that siRNA-mediated depletion of VAPB or PTPIP51 disrupts synaptic activity, leading to alterations in synaptic vesicle release and dendritic spine numbers—likely due to impaired Ca^2+^ homeostasis and mitochondrial ATP production, both of which are key functions of VAPB at these sites [28].

In summary, while earlier studies established that VAPB mutation or loss impairs autophagy and that VAPB overexpression can be neuroprotective, but the major advance in this study is defining how VAPB is redistributed in human ALS tissue across multiple disease subtypes and linking this to selective neuronal vulnerability. We show that MNs retaining soluble VAPB are relatively resistant to aggregate pathology, whereas vulnerable neurons exhibit marked VAPB depletion together with sequestration into TDP-43, FUS, and DPR-associated inclusions. In addition, VAPB accumulates at enlarged C-bouton–associated compartments with Sigma-1 receptor, suggesting a compartment-specific role in synaptic stress adaptation. Together, human neuropathology, iPSC-derived MNs, and autophagy assays support a model in which VAPB depletion impairs autophagic turnover, promotes aggregate persistence, and drives selective MN degeneration.

## Supplementary Information

Additional file1 (DOCX 839 KB)

Additional file2 (DOCX 21 KB)

Additional file3 (DOC 82 KB)

Additional file4 (DOCX 19 KB)

## Data Availability

Available promptly upon request
