# Supplementary material for: VAPB confers selective neuroprotection by driving autophagic degradation of pathogenic aggregates in ALS
Source: Acta Neuropathol Commun. 2026 May 29;14:127. doi: 10.1186/s40478-026-02298-8 (PMC13255306; doi:10.1186/s40478-026-02298-8)
Supplement: Supplementary file 1 — Additional file1 (DOCX 839 KB) [file 40478_2026_2298_MOESM1_ESM.docx]

## Supporting Figure 1


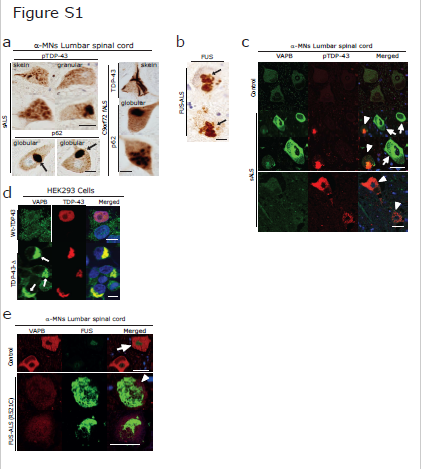


**(a-b)** DAB immunohistochemistry performed on sALS, C9orf72 fALS, and FUS ALS lumbar spinal cord α-MNs, showing various morphologies of pTDP-43, p62, and **(b)** FUS aggregates, scale bars: 50 µm.

**(c)** Double immunofluorescence labeling was performed on control and sALS lumbar spinal cord α-MNs using VAPB and pTDP-43 antibodies. The results show reduced levels of VAPB in the presence pTDP-43 aggregates (white arrowheads), while increased levels of VAPB in the MNs were associated with absence of TDP-43 aggregates (white arrows) in sALS lumbar spinal cord α-MNs. Scale bars: 50 µm. Three sections each were analyzed from sALS patients (n=7) and age-matched normal individuals (n=4).

**(d)** VAPB immunolabelling performed on HEK293 cells overexpressing either the Wt TDP-43 (upper panel) or the mutant TDP43-delta (lower panel) showing the aggregation (arrows) and sequestration of endogenous VAPB together with the aggregates of mutant TDP-43. Scale bars: 10 µm.

**(e)** Double immunofluorescence labeling was performed on control and FUS-ALS lumbar spinal cord α-MNs using VAPB and FUS antibodies. The results show reduced levels of VAPB in the presence FUS aggregates (white arrowheads). Three sections each were analyzed from FUS-ALS patients. Scale bars: 50 µm. Three sections each were analyzed from FUS-ALS patients (n=3) and age-matched normal individuals (n=3).

## Supporting Figure 2


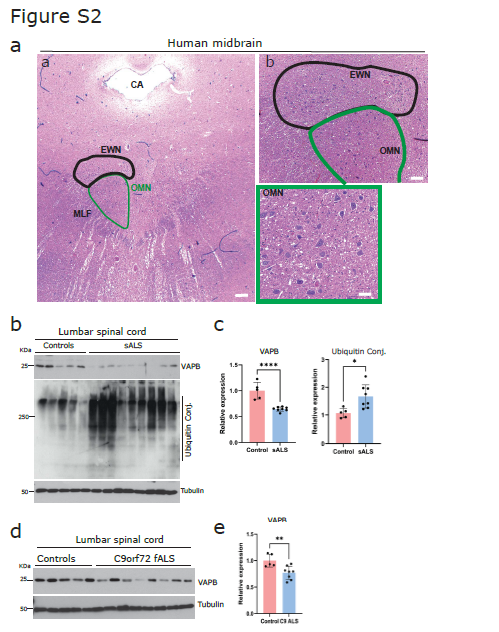


**(a)** Hematoxylin-eosin (H&E)-stained control midbrain paraffin sections showing the region of oculomotor neurons (left, green circle) and enlarged view (right). CA- Cerebral Aqueduct, EWN-Edinger Westphal nucleus, OMN- Oculomotor nucleus, MLF- Medial longitudinal fasciculus. Scale bars: a:600 µm, b:300µm, c: 200µm.

**(b-d)** Immunoblot analysis showed a decreased VAPB level in sALS **(b)** and in C9orf72-fALS **(d).** Corresponding densitometric data represents the relative band intensity of Western blot analysis **(c, e).** Tubulin was used as a loading control. Statistical analyses were done using GraphPad Prism software. Student's t-test for comparison between two groups. ns= not significant; * = p-value lower than 0.05; ** = p-value lower than 0.01; **** = p-value lower than 0.0001. Values were expressed as mean ± SD from three independent blots. A. U= Arbitrary units).

## Supporting Figure 3


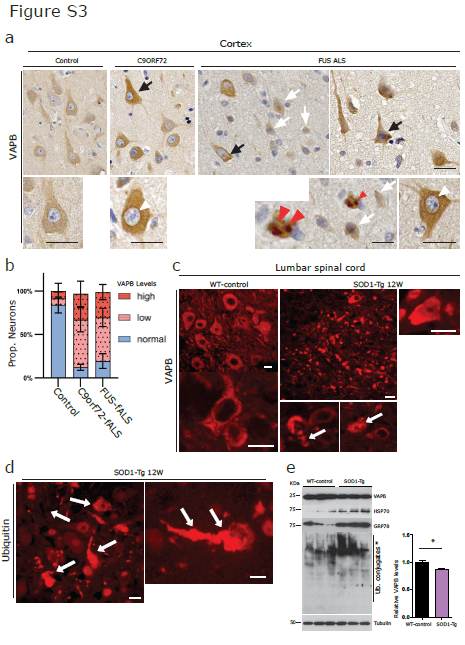


**(a-b)** DAB immunohistochemistry performed on C9orf72 fALS and FUS-ALS cortex showing various patterns of VAPB immunoreactivity. Note the strong nuclear envelope immunoreactivity (white arrowheads), increased cytoplasmic immunoreactivity (black arrows), reduced cytoplasmic immunoreactivity (white arrows), and VAPB accumulation (red arrowhead). Representative images from three sections were analyzed from C9orf72 fALS patients (n= 4), and FUS-ALS patients (n= 3) compared to the age-matched control (n=4). Scale bars: 20 µm. Quantification of the VAPB levels **(b)**

**(c-d)** Immunofluorescence labeling using VAPB **(b)** and **(c)** Ubiquitin antibodies showing accumulations of VAPB (arrows in b) as well as of Ubiquitin (arrows in c) in the remaining MNs of 12 weeks SOD1-G93A lumbar spinal cord, compared to age-matched normal controls. Note the massive loss of MNs in the lumbar spinal cord of SOD1 mice at 12 weeks. Representative images from one of three sections analyzed from G93A SOD1 mice (n= 3, 12 weeks), compared to the wild-type littermates (n=3,12 weeks). Scale bars: 50 µm.

**(e)** Immunoblot analysis shows decreased VAPB levels in the SOD1-G93A lumbar spinal cord compared to Wt controls. Statistical analyses were done using GraphPad Prism software. Student's t-test for comparison between two groups. ns= not significant; * = p-value lower than 0.05. Values were expressed as mean ± SD from three independent blots. A. U= Arbitrary units).
