# Supplementary material for: VAPB confers selective neuroprotection by driving autophagic degradation of pathogenic aggregates in ALS
Source: Acta Neuropathol Commun. 2026 May 29;14:127. doi: 10.1186/s40478-026-02298-8 (PMC13255306; doi:10.1186/s40478-026-02298-8)
Supplement: Supplementary file 2 — Additional file2 (DOCX 21 KB) [file 40478_2026_2298_MOESM2_ESM.docx]

| **Case No.** | **Age** | **Gender** | **Cause of death** | **PMI (hrs)** | **Clinical diagnosis** | **Pathological**  **diagnosis** | **pTDP-43** |
| --- | --- | --- | --- | --- | --- | --- | --- |
| 1 | 68 | M | euthanasia | 12 | fALS | ***C9orf72*** | ++ |
| 2 | 69 | M | Muscle wasting | 26 | fALS | ***C9orf72*** | +++ |
| 3 | 61 | M | unknown | 24 | fALS | ***C9orf72*** | ++ |
| 4 | 51 | M | pneumonia | 12 | fALS | ***C9orf72*** | ++ |
| 5 | 64 | F | respiratory | 24 | fALS | ***C9orf72*** | +++ |
|  |  |  |  |  |  |  |  |
| 1 | 40 | M | unknown | 12 | R521C fALS | ***FUS*** | - |
| 2 | 35 | F | pneumonia | 12 | R521C fALS | ***FUS*** | - |
| 3 | 40 | F | respiratory | 12 | R521C fALS | ***FUS*** | - |
| 4 | 40 | F | respiratory | 26 | R521C fALS | ***FUS*** | - |
|  |  |  |  |  |  |  |  |
| 1 | 68 | M | Muscle wasting | 24 | sALS | sALS | +++ |
| 2 | 43 | M | Respiratory | 12 | sALS | sALS | +++ |
| 3 | 75 | F | Muscle wasting | 36 | sALS | sALS | +++ |
| 4 | 70 | F | Muscle wasting | 24 | sALS | sALS | ++ |
| 5 | 64 | M | Urosepsis | 24 | sALS | sALS | +++ |
| 6 | 58 | M | pneumonia | 12 | sALS | sALS | +++ |
| 7 | 65 | F | Respiratory | 24 | sALS | sALS | +++ |
| 8 | 76 | F | Muscle wasting | 12 | sALS | sALS | +++ |
| 9 | 68 | F | pneumonia | 24 | sALS | sALS | +++ |
|  |  |  |  |  |  |  |  |
| 1 | 54 | M | Sepsis | 15 | - | ***Normal*** | - |
| 2 | 70 | M | Heart failure | 7 | - | ***Normal*** | - |
| 3 | 81 | M | Organ failure | 16 | - | ***Normal*** | - |
| 4 | 54 | M | Sepsis | 15 | - | ***Normal*** | - |

**Supplementary Table 1.** Patients examined in this study. PMI = Postmortem interval

**Overall TDP-43 load/Immunoreactivity in MNs**

**+++:** (60-70 %) MNs showing TDP43 immunoreactivity, strong.

**++ :** 30-60% MNs showing TDP43 immunoreactivity, medium.

**+ :** below 30 % MNs showing TDP43 immunoreactivity, mild.
