# Supplementary material for: VAPB confers selective neuroprotection by driving autophagic degradation of pathogenic aggregates in ALS
Source: Acta Neuropathol Commun. 2026 May 29;14:127. doi: 10.1186/s40478-026-02298-8 (PMC13255306; doi:10.1186/s40478-026-02298-8)
Supplement: Supplementary file 3 — Additional file3 (DOC 82 KB) [file 40478_2026_2298_MOESM3_ESM.doc]

**Supplementary Table 2:** List of primary and secondary antibodies used in this study
 (Previously used by us in the references: **[1-5]**

| **Antibody** | **Commercial source** | **Species** | **Reference** | **Working Dilution** | | |
| --- | --- | --- | --- | --- | --- | --- |
|  |  |  |  | **IHC/IF** | | **WB** |
| **Primary antibody** | | | | | | |
|  |  |  |  |  |  | |
| Anti-GADD | Santa Cruz | Rabbit | SC-793 | 1:100 | 1:1000 | |
| Anti-GRP78 (Bip) | BD Biosciences | Mouse | 610978 | 1:100 | 1:1000 | |
| Anti-p62 | MBL | Rabbit | PM045 | 1:100 | 1:50,000 | |
| Anti-p62 | Sigma Aldrich | Rabbit | P0067 | 1:100 | 1:50,000 | |
| Anti-LC3 | Sigma Aldrich | Rabbit | L7543 | 1:100 | 1:50,000 | |
| Αnti α-Tubulin | Sigma Aldrich | Mouse | T5168 | **-** | 1:10,000 | |
| Anti-VAPB | Homemade | Rabbit | **-** | 1:100 | 1:1000 | |
| Anti-ULK-1 | Sigma Aldrich | Rabbit | HPA063990 | 1:100 | 1:1000 | |
| Anti-SigR1 | Proteintech | Rabbit | 15168-1-AP | 1:100 | **-** | |
| Anti-SigR1 | Santa Cruz | Mouse | Sc-166392 | 1:50 | 1:1000 | |
| Anti-Ubiquitin (ub) | Dako | Rabbit | Z0458 | 1:100 | 1:1000 | |
| Anti-pTDP43 | Cosmo Bio Co. LTD | Mouse | TIP-PTD-M01 | 1:5000 | **-** | |
| Anti-Fus | Novus Biologicals | Rabbit | NB100-2599 | 1:100 | 1:50,000 | |
| Anti-TIAR1 | BD Biosciences | Mouse | 610352 | 1:100 | 1:1000 | |
| Anti-STX17 | Sigma Aldrich | rabbit | HPA001204 | 1:100 | 1:1000 | |
| Anti-ATG5 | Novus | rabbit | NB110-53818 | 1:100 | 1:1000 | |
| Poly HRP-GAMs/Rb IgG | Immunologic a VWR | Ms/Rb | VWRKDPVB500HRP | Read-to-use |  | |
| Poly HRP-Anti Goat IgG | Immunologic a VWR | Goat | VWRKDPVG110HRP | Read-to-use |  | |
|  |  |  |  |  |  | |
| Alexa Fluor 488 goat anti-mouse | Life Technologies | Goat | A11001 | 1:500 |  | |
| Alexa Fluor 555 goat anti-mouse | Life Technologies | Goat | A21424 | 1:500 |  | |
| Alexa Fluor 488 goat anti-rabbit | Life Technologies | Goat | A11008 | 1:500 |  | |
| Alexa Fluor 546 goat anti-rabbit | Life Technologies | Goat | A11010 | 1:500 |  | |
| Alexa Fluor 488 donkey anti-goat | Life Technologies | Donkey | A11055 | 1:500 |  | |
| Goat anti-rabbit IgG (H+L), HRP | Thermo Scientific | Goat | 31460 |  | 1:10000 | |

**REFERENCES**

1 Prause J, Goswami A, Katona I, Roos A, Schnizler M, Bushuven E, Dreier A, Buchkremer S, Johann S, Beyer C, Deschauer M, Troost D, Weis J. Altered localization, abnormal modification and loss of function of Sigma receptor-1 in amyotrophic lateral sclerosis. Hum Mol Genet 2013; 22: 1581-600

2 Vollrath JT, Sechi A, Dreser A, Katona I, Wiemuth D, Vervoorts J, Dohmen M, Chandrasekar A, Prause J, Brauers E, Jesse CM, Weis J, Goswami A. Loss of function of the ALS protein SigR1 leads to ER pathology associated with defective autophagy and lipid raft disturbances. Cell Death Dis 2014; 12: 243

3 Dreser A, Vollrath JT, Sechi A, Johann S, Roos A, Yamoah A, Katona I, Bohlega S, Wiemuth D, Tian Y, Schmidt A, Vervoorts J, Dohmen M, Beyer C, Anink J, Aronica E, Troost D, Weis J, Goswami A. The ALS-linked E102Q mutation in Sigma receptor-1 leads to ER stress-mediated defects in protein homeostasis and dysregulation of RNA-binding proteins. Cell Death Differ 2017; 24: 1655-71

4 Tripathi P, Guo H, Dreser A, Yamoah A, Sechi A, Jesse CM, Katona I, Doukas P, Nikolin S, Ernst S, Aronica E, Glass H, Hermann A, Steinbusch H, Feller AC, Bergmann M, Jaarsma D, Weis J, Goswami A. Pathomechanisms of ALS8: altered autophagy and defective RNA binding protein (RBP) homeostasis due to the VAPB P56S mutation. Cell Death Dis 2021; 12: 466

5 Jesse CM, Bushuven E, Tripathi P, Chandrasekar A, Simon CM, Drepper C, Yamoah A, Dreser A, Katona I, Johann S, Beyer C, Wagner S, Grond M, Nikolin S, Anink J, Troost D, Sendtner M, Goswami A, Weis J. ALS-Associated Endoplasmic Reticulum Proteins in Denervated Skeletal Muscle: Implications for Motor Neuron Disease Pathology. Brain Pathol 2017; 27: 781-94
