# Supplementary material for: VAPB confers selective neuroprotection by driving autophagic degradation of pathogenic aggregates in ALS
Source: Acta Neuropathol Commun. 2026 May 29;14:127. doi: 10.1186/s40478-026-02298-8 (PMC13255306; doi:10.1186/s40478-026-02298-8)
Supplement: Supplementary file 4 — Additional file4 (DOCX 19 KB) [file 40478_2026_2298_MOESM4_ESM.docx]

**Table S3 Semi quantitative analysis**

**Figure 3g-h**

|  | Total number of neurons counted | Total number of aggregates | Total no. Colocalization | Colocalization  (%) |
| --- | --- | --- | --- | --- |
|  |  |  |  |  |
| **VAPB+GA** | 110 | 48 | 29 | 60.41 |
|  |  |  |  |  |
| VAPB+TDP43 | 91 | 32 | 19 | 48.71 |
|  |  |  |  |  |
|  |  |  |  |  |

|  | Total number of cells counted | Total number of aggregates | Total no. Colocalization | Colocalization  (%) |
| --- | --- | --- | --- | --- |
|  |  |  |  |  |
| **VAPB+FUS** | **62** | **29** | **13** | **44.82** |
|  |  |  |  |  |

**Figure 4a**

**Figure 4C**

|  | Total number of MNs counted | Total number of aggregates | Total no. Colocalization | Colocalization  (%) |
| --- | --- | --- | --- | --- |
|  |  |  |  |  |
| **EGFP+FUS** | **49** | **22** | **17** | **77.27** |
|  |  |  |  |  |

**Figure 4D**

|  | Total number of MNs counted | Total number of aggregates | Total no. Colocalization | Colocalization  (%) |
| --- | --- | --- | --- | --- |
|  |  |  |  |  |
| **VAPB+FUS** | **35** | **20** | **12** | **60%** |
|  |  |  |  |  |

|  | Total number of cells counted | Total number of TIA SGs | Total no. Colocalization | Colocalization  (%) |
| --- | --- | --- | --- | --- |
|  |  |  |  |  |
| **VAPB+TIA1** | **48** | **66** | **24** | **36.36** |
|  |  |  |  |  |

**Figure 4E**

**Figure S3**

Aggregated structures are very rare to be seen in DAB

|  | **VAPB** | Total number with high levels | Total number with low levels | normal | VAPB Aggregated |
| --- | --- | --- | --- | --- | --- |
| **Controls** | **149** | **12** | **10** |  |  |
| **C9orf72 fals** | **138** | **42** | **74** | 15 | 5 |
| **FUS-ALS** | **115** | **34** | **59** | 22 | 7 |

|  | Total number of cells counted | Total number of aggregates | Total no. Colocalization | Colocalization  (%) |
| --- | --- | --- | --- | --- |
|  |  |  |  |  |
| **VAPB+TDP43** | **55** | **35** | **22** | **62%** |
|  |  |  |  |  |

**Figure S1 d**
